# Iron Binding at Specific Sites within the Octameric HbpS Protects Streptomycetes from Iron-Mediated Oxidative Stress

**DOI:** 10.1371/journal.pone.0071579

**Published:** 2013-08-27

**Authors:** Ina Wedderhoff, Inari Kursula, Matthew R. Groves, Darío Ortiz de Orué Lucana

**Affiliations:** 1 Department of Biology/Chemistry, University of Osnabrueck, Osnabrueck, Germany; 2 Centre for Structural Systems Biology at the German Electron Synchrotron, Helmholtz Centre for Infection Research, Hamburg, Germany; 3 Department of Biochemistry, University of Oulu, Oulu, Finland; 4 Department of Pharmacy, University of Groningen, Groningen, The Netherlands; Arizona State University, United States of America

## Abstract

The soil bacterium *Streptomyces reticuli* secretes the octameric protein HbpS that acts as a sensory component of the redox-signalling pathway HbpS-SenS-SenR. This system modulates a genetic response on iron- and haem-mediated oxidative stress. Moreover, HbpS alone provides this bacterium with a defence mechanism to the presence of high concentrations of iron ions and haem. While the protection against haem has been related to its haem-binding and haem-degrading activity, the interaction with iron has not been studied in detail. In this work, we biochemically analyzed the iron-binding activity of a set of generated HbpS mutant proteins and present evidence showing the involvement of one internal and two exposed D/EXXE motifs in binding of high quantities of ferrous iron, with the internal E_78_XXE_81_ displaying the tightest binding. We additionally show that HbpS is able to oxidize ferrous to ferric iron ions. Based on the crystal structure of both the wild-type and the mutant HbpS-D_78_XXD_81_, we conclude that the local arrangement of the side chains from the glutamates in E_78_XXE_81_ within the octameric assembly is a pre-requisite for interaction with iron. The data obtained led us to propose that the exposed and the internal motif build a highly specific route that is involved in the transport of high quantities of iron ions into the core of the HbpS octamer. Furthermore, physiological studies using *Streptomyces* transformants secreting either wild-type or HbpS mutant proteins and different redox-cycling compounds led us to conclude that the iron-sequestering activity of HbpS protects these soil bacteria from the hazardous side effects of peroxide- and iron-based oxidative stress.

## Introduction

Streptomycetes are highly abundant in soils and decaying vegetation. In such ecological niches, they have to co-exist with other organisms (i.e. a number of bacteria, fungi, plants or insects) and need to respond to variable conditions (i.e. changes in environmental pH, humidity, salinity, osmotic pressure, the presence or absence of nutrient sources or redox state) [1], [2], [3]. Redox reactions events are often related to the presence of iron that is the second most abundant metal and the fourth most abundant element in the earth's crust. Iron is found in various oxidation states (from −II to +VI), but the most common are II (Fe^2+^; ferrous form) and III (Fe^3+^; ferric form). Under physiological conditions, Fe^3+^ is more abundant than Fe^2+^, which in contrast to Fe^3+^ is highly soluble in water. Given that the redox potential of Fe^2+^/Fe^3+^ is extremely variable (from −300 to +700 mV), iron behaves as a versatile prosthetic protein component that alone, or as part of Fe-S clusters or haem, plays a crucial role in many biological processes (e.g. oxygen transport, electron transfer or regulatory cascades) and is essential for nearly all known organisms [4], [5], [6].

Despite these favourable properties, high concentrations of iron ions can be toxic. In the presence of H_2_O_2_, Fe^2+^ participates in the generation of highly reactive hydroxyl radicals *via* the Fenton reaction (Fe^2+^+H_2_O_2_→Fe^3+^+**^•^OH**+^−^OH). In general, reactive oxygen species (ROS, including free radicals, oxides and peroxides) provoke damage in proteins, leading to a variety of oxidative modifications (i.e. hydroxylation of aromatic groups and aliphatic amino acid side-chains, formation of dityrosines and disulfides, and conversion of certain amino acid residues to carbonyl derivatives). These modifications are often irreversible and may result in aggregation and/or degradation [7]. Importantly, metal-dependent generated ^•^OH react at specific sites within or in proximal vicinity of the metal-binding site [8]. ROS-mediated oxidation of lipids, termed lipid peroxidation, induces oxidation of membrane proteins as well as disturbances within membranes (i.e. alteration of their integrity, fluidity and permeability) that lead to cell dysfunction [9], [10]. During oxidation, polyunsaturated fatty acids are degraded to a variety of products including highly reactive aldehydes (e.g. malondialdehyde and 4-hydroxynonenal) [11]. In contrast to ^•^OH-dependent local oxidation of proteins, aldehydes diffuse throughout the cell, resulting in oxidative modification of proteins as well as DNA and RNA that are located far from the initial point of oxidative attack [12].

In order to counteract the hazardous properties of peroxides and iron ions, soil bacteria as well as other organisms use different protective systems. Hydrogen peroxide that is continuously produced during respiration can be scavenged by a variety of enzymes, including catalases, various peroxidases and other H_2_O_2_-scavenging proteins; these have diverse kinetic optima or protein stability that are required for correct function during growth under changing environmental conditions [13]. Iron ions can be complexed by siderophores or sequestered and stored by ferritins or Dps proteins [14], [15]. For instance, ferritins that are either haem-free or contain a haem (bacterioferritins), oxidize Fe^2+^ to Fe^3+^ within a so-called ferroxidase center that comprises several glutamate, aspartate and histidine residues [16], [17]. The non-toxic Fe^3+^ can be subsequently stored in so-termed protein nanocages [18], [19]. Ferritins and bacterioferritins have a hollow and roughly spherical construction (∼450 kDa) comprising 24 subunits with an iron storage cavity in which up to 4500 iron ions can be accommodated [14]. Haem in bacterioferritins is assumed to mediate iron-core reduction and iron release [20], [21].

Iron binding by proteins takes place at specific sites, here defined as iron-binding motifs. Some of these motifs include positive charged (histidine, lysine and arginine), negative charged (glutamic acid and aspartic acid) as well as cysteine and tyrosine residues. Iron in haem or iron-sulfur clusters can be coordinated *via* a CXXCH binding motif (i.e. in the apo-cytochrome C) or CXC and CXXC motifs (i.e. in the nitrogen fixation-related protein NifU) [22], [23]. The iron-sulfur cluster assembly repressor IscR in *E. coli* binds iron *via* three cysteine residues and one histidine residue in a CX_5_CX_5_CX_2_H motif [24]. The mononuclear non-haem iron (II) oxygenases including the extradiol-cleaving catechol dioxygenase BphC, naphthalene dioxygenase NDO and phenylalanine hydroxylase PheH bind a Fe^2+^ iron ion *via* a well conserved motif consisting of two histidines and one glutamate or aspartate residue [25]. Glutamates and aspartates within D/EXXE motifs have also been implicated in binding and transport of iron ions in the permease Ftr1p from *Saccharomyces cerevisiae* and PmrB from the two-component system PmrA/PmrB of *Salmonella enterica* serovar Typhimurium [26], [27]. Interestingly, some of the glutamate residues involved in interactions with iron ions in ferritins and Dps proteins are located within EXXE motifs [28], [29].

The production of some iron-complexing molecules (i.e. siderophores or ferritins) are under the control of the ferric uptake regulator (Fur) protein [5]. Noteworthy, the expression of Fur is also regulated by OxyR and SoxRS that control the expression of anti-oxidative stress genes [30]. A Fur-like protein, namely FurS from streptomycetes, regulates the transcription of the catalase-peroxidase CpeB; *furS* and *cpeB* form an operon [31]. Recent studies demonstrated that *furS*-*cpeB* is under the control of the three-component system HbpS-SenS-SenR in which the extracellular protein HbpS acts as an accessory element of the two-component system SenS-SenR [32]. Protein-DNA interaction studies revealed that the phosphorylated response regulator SenR activates the transcription of *furS*, *cpeB*, *hbpS*, *senS* and *senR* [32], [33]. HbpS is a novel type of haem-binding protein and is secreted *via* the twin-arginine translocation (Tat) pathway [34], [35]. High resolution 3D crystal structures (PDB: 3FPV and 3FPW) of HbpS reveal an octameric assembly; analyses of electron density maps followed by mutagenesis and biochemical studies showed that a serine (Ser-26) and a histidine (His-28) within the N-terminal domains from neighboring subunits are crucial for oligomerization as well as for interaction with SenS-SenR [36]. Biochemical studies revealed that haem/iron-free HbpS inhibits the autophosphorylation of the sensor kinase SenS under non-oxidative stress conditions. However, in the presence of iron/haem-mediated oxidative stress HbpS significantly enhances SenS autophosphorylation [36], [37]. Analysis of the crystal structure obtained in the presence of haemin (PDB: 3FPW) revealed the presence of an iron ion interacting with exposed lysine residues at position 108 (Lys-108) on the surface of the octamer. Further studies revealed that HbpS is able to degrade haem, leading to the assumption that the released iron from haem can be captured by Lys-108 [36]. Notably, the HbpS-K108A mutant protein is not able to mediate the haem-based activation of SenS autophosphorylation. The inactive form of SenS lacks iron and its activation is iron-dependent [37]. It remains to be elucidated to what extent the iron ion at Lys-108 modulates the phosphorylation state of the membrane-embedded sensor kinase SenS. Furthermore, fluorescence resonance energy transfer (FRET), circular dichroism (CD) and electron paramagnetic resonance (EPR) spectroscopic studies showed that under iron-mediated oxidative stress HbpS undergoes oxidative modifications (i.e. dityrosine formation) that are accompanied by overall conformational changes [38], [39]. These are proposed to be involved in the activation of SenS during oxidative stress. Remarkably, dityrosine formation occurred at specific sites within the HbpS octamer [38], [40], leading to the assumption that these sites are located in the proximal vicinity to an iron-binding site that remains to be unambiguously identified.

Physiological studies using a *Streptomyces reticuli* mutant lacking chromosomal *hbpS* showed that this mutant strain exhibits an increased sensitivity to haem as well as iron ions [34], [37]. Because HbpS degrades haem *in vitro* and *in vivo*, we have suggested that this process provides *S. reticuli* with protection against haem toxicity [35], [36]. The cleavage of haem by other proteins (i.e. haem oxygenase from *Corynebacterium diphtheriae*) results in the conversion products biliverdin and CO with the release of iron ions [41]. Amino acid sequence comparisons of HbpS yielded a large number of putative homologues encoded within genomes of some Gram-positive bacteria. These include several *Streptomyces sp*. (i.e. *S. coelicolor* A3 (2), *S. kasugaensis*, *S. lividans* and *S. griseus*) and other actinobacteria (i.e. *Arthrobacter aurescens, Rhodococcus sp.* RH1, *Nocardia cyriacigeorgica* GUH-2 and *Leifsonia xyli*). Other homologues were recorded by Gram-negative bacteria including *Vibrio cholera*, *Klebsiella pneumoniae*, *Pseudomonas putida* G7, *Sphingomonas aromaticivorans* or *Agrobacterium tumefaciens* C58. Interestingly, some of the *hbpS*-like genes are located within operons encoding proteins that degrade aromatic and non-aromatic compounds [42]. Further comparisons revealed that the main portion of HbpS and all HbpS-like proteins consist of the so-called DUF336 domain [43]. This domain is being postulated to participate in cofactor binding or to have enzymatic activity (Pfam: PF03928). The full details of the molecular pathway whereby HbpS mediates resistance to iron ions remain to be elucidated.

Analyses of the crystal structure of HbpS and our previously obtained data led to the assumption that Glu-78 and Glu-81, forming an EXXE motif, might be involved in iron binding [38], [40]. Further analyses revealed that HbpS contains in total three D/EXXE motifs with conserved surrounding amino acids. In this work, we generated a set of mutant HbpS proteins with exchanged E or D within these putative iron-binding motifs and surrounding amino acids as well. Subsequent biochemical studies allowed characterization of their iron-binding activities. Furthermore, the crystal structure of an HbpS mutant was solved to obtain detailed insights into the major iron-binding site. Finally, a set of *Streptomyces* transformants was generated in order to investigate the role of the HbpS-iron complex during oxidative stress *in vivo*.

## Materials and Methods

### Bacterial strains, growth conditions and preparation of spores

*E. coli* strains BL21 (DE3) pLysS and DH5α were cultivated in LB medium. *Streptomyces lividans* 66 (*S. lividans*; D.A. Hopwood, John Innes Institute, Norwich, UK) and its transformants were cultivated in complete (R2) or minimal liquid media supplemented with the indicated carbon source [44]. To gain spore suspensions, R2 agar plates containing soja and mannitol were used. Plates containing *Streptomyces* spores were flooded with sterile, distilled water, and the resulting spore suspension was filtered [44]. After filtration spore suspensions were washed twice with sterile, distilled water and subsequently stored in 50% (v/v) glycerol. Estimation of spore concentrations was performed by counting using a thoma chamber. The results obtained were confirmed by quantification of colonies after the spores had been spread and grown on R2 plates [44].

### Chemicals and enzymes

Chemicals for SDS-PAGE and native-PAGE were obtained from ROTH. Plumbagin, hydrogen peroxide (H_2_O_2_), haemin, potassium ferrocyanide, 3,3′-diaminobenzidine tetrahydrochloride were supplied by Sigma. Iron ion salts were purchased from Merck. Molecular weight DNA markers, restriction enzymes, T4 Ligase and DNA polymerase for PCR were obtained from Thermo Scientific or New England Biolabs.

### Isolation of DNA and transformation

Isolation of plasmids and extraction of DNA from gels were performed using the Qiagen mini and midi plasmid preparation and the gel extraction kits. Plasmids were used to transform *E. coli* DH5α by electroporation and/or *E. coli* BL21 (DE3) pLysS by the calcium chloride method. Isolation of *S. lividans* protoplasts and their transformation with plasmids were done as described [44]. *S. lividans* transformants were selected using an overlay of 2.5 ml 0.4% agarose containing 200 µg/ml thiostrepton [44].

### Site-directed mutagenesis

To generate point mutations in *hbpS*, the pETHbpS plasmid (Table S1) was used as a template for single or two step PCR reactions, followed by restriction and ligation. For design of following mutants single step PCR with subsequent primers (Table S2) was applied: HbpS-E78A: PE78A and PET11Rev; HbpS-E81A: PE81A and PET11Rev; HbpS-E78D: PE78D and PET11Rev; HbpS-E81D: PE81D and PET11Rev; HbpS-E78D/E81D: PEEDD and PET11Rev; HbpS-R82A: PER82A and PET11Rev; HbpS-K83A: PK83A and PET11Rev; HbpS-K83R: PK83R and PET11Rev; HbpS-E78A/E81A: PEAFor and PET11Rev (as template for PCR, pETHbpS-E78A was used). Each of the resulting PCR fragments was cleaved with *Bsi*WI and *Hin*dIII and subsequently ligated with the longer *Bsi*WI-*Hin*dIII fragment of pETHbpS.

To obtain HbpS-D141A, HbpS-E144A and HbpS-D143A the primer PET11For was used in combination with either PD141ARev or PD143Rev or PE144ARev for PCR. The resulting fragments as well as the vector pETHbpS were cut with *Nco*I and *Hin*dIII and were ligated afterwards. To obtain HbpS-E43A, the primers PET11For and PRevE43A were used; the PCR fragment was cleaved with *Pml*I and *Nco*I and subsequently ligated with the longer *Pml*I-*Nco*I fragment of pETHbpS.

The two-step PCR technique was used to obtain HbpS-E46A, HbpS-E43A/E46A and HbpS-Y77A. In the first step, pETHbpS was used as template for PCR. The reactions additionally contained flanking primers PET11For and PET11Rev as well as overlapping primers PFor46A and PRev46A (for HbpSE46A), PFor43-46 and PRev43-46 (for HbpSE43A/E46A), and PYAFor2 and PYARev1 (for HbpSY77A). In the second step, the obtained PCR products (as template) and the flanking primers were used. The resulting fragments and the vector pETM11 were cut with *Nco*I and *Hin*dIII and subsequently ligated.

Each of the ligation products were used to transform *E. coli* DH5α and the correctness of the introduced mutations was analyzed by sequencing. To get recombinant HbpS proteins, the pETHbpS plasmids were used to transform *E. coli* BL21 (DE3) pLysS. Plasmids as well as oligonucleotides used in this work are listed in Table S1 and Table S2, respectively.

### Cloning of *hbpS* genes in *Streptomyces*

To clone *hbpS* wild-type as well as the generated *hbpS* mutants (E78A, E81A and E78A/E81A) in *Streptomyces* the plasmids pUC18, pUKS13 and pWHM3 were used. The pUKS13 plasmid contains the *furS*-*cpeB* operon with its promoter region. *furS* in pUKS13 is mutated and encodes the protein FurSY59F that has lost its transcriptional repressor activity [45]. The smaller *Eco*RI-*Stu*I fagment from pUKS13 was ligated with the longer *Eco*RI-*Sma*I fragment from pUC18, leading to the construct pUKS20. An *Nco*I restriction site was then introduced between the ribosomal-binding site and the start codon of *cpeB* by using pUKS20 as a template for PCR and following primers: For fragment A: PANotFor located at the upstream region of *furS* and PANcoRev located between *furS* and *cpeB*; for fragment B: PBNcoFor located between *furS* and *cpeB* and PBHinRev located at the downstream region of the truncated *cpeB* gene. Fragment A was cut with *Not*I and *Nco*I and Fragment B was cut with *Nco*I and *Hin*dIII. Both fragments were ligated to the longer *Not*I-*Hin*dIII fragment from pUKS20, leading to the construct pUKS21. The insertion of *Nco*I was analysed by sequencing. The complete *hbpS* gene, also containing the sequence for the signal peptide, was amplified using the plasmid pUKS10 [46] as a template for PCR and following primers: PHbpFor and PHbpRev. The resulting amplicon was cut with *Nco*I and *Hin*dIII and ligated with the longer *Nco*I-*Hin*dIII fragment from pUKS21, leading to the construct pUKS22. This contained *furS* and *hbpS* in one operon, a functional promoter for *streptomycetes* and an active ribosomal-binding site in front of *hbpS*. Most importantly, the encoded FurS protein is inactive and therefore not able to repress the transcription of *furS*-*hbpS*.

To clone the *hbpS* mutant genes as well as the wild-type in *Streptomyces*, the *Pml*I-*Hin*dIII smaller fragment (containing the DNA region with the corresponding mutation) from pETHbpS-E78A, pETHbpS-E81A and pETHbpS-E78A/E81A was at first ligated with the longer *Pml*I-*Hin*dIII fragment from pUKS22, leading to the plasmid constructs pUKS23, pUKS24 and pUKS25, respectively. The *Eco*RI-*Hin*dIII fragment from pUKS22 (with the *hbpS* WT gene), pUKS23 (with the E78A codon), pUKS24 (with the E81A codon) and pUKS25 (with the codons E78A/E81A), respectively, was ligated with the longer *Eco*RI-*Hin*dIII fragment from pWHM3. All ligation products were introduced into *E. coli* DH5α by electroporation. Subsequently, each of the plasmid constructs was analyzed with restriction enzymes and/or by sequencing. The resulting plasmid constructs pWHbpS, pWHbpS-E78A, pWHbpS-E81A and pWHbpS-E78A/E81A were used to transform *S. lividans* protoplasts.

### SDS-PAGE and Western blotting

Native- and SDS-PAGE were performed as described [47]. For Western blotting, proteins were separated by 12% SDS-PAGE and transferred to a PVDF membrane [48], which was blocked for 1 h at room temperature with PBS containing 5% skimmed milk powder, and subsequently incubated overnight at 4°C with previously described *anti*-HbpS antibodies [34]. After treatment with secondary *anti*-guinea pig antibodies conjugated with alkaline phosphatase, the membrane was stained with 5-bromo-4-chloro-3-indolyl-phosphate and nitroblue tetrazolium.

### Detection of HbpS proteins in *S. lividans* transformants

*S. lividans* transformants containing pWHbpS, pWHbpS-E78A, pWHbpS-E81A or pWHbpS-E78A/E81A as well as the host strain *S. lividans* (without any plasmid) were cultivated in complete medium and subsequently in minimal medium containing 0.25% yeast extract and incubated at 30°C as described earlier [34]. Mycelia were centrifuged and the supernatant was subsequently filtrated. Proteins from the filtrate were subjected to a fractionated ammonium sulfate precipitation. The 60%–90% protein fraction was subjected to SDS-PAGE followed by Western blot using *anti*-HbpS antibodies.

### Growth assays

#### Microscopical studies

*S. lividans* spores (each 1×10^5^) from the host strain and the transformants containing pWHbpS, pWHbpS-E78A, pWHbpS-E81A or pWHbpS-E78A/E81A were inoculated with 500 µl R2 medium on a microtiter plate (12-well TC-plates, growth area: 3.66 cm^2^, volume: 6.30 ml, dimension:128×85×22 mm) and incubated at 30°C. The cultivation medium was supplemented without or with 0.0025% H_2_O_2_. Growth was analysed for several days under the microscope (Axio observer Z1 inverse microscope, Zeiss) with four times magnification.

#### Growth on plates

R2 agar plates lacking or containing 0.0025% H_2_O_2_, or 0.005 mM plumbagin were used. One µl of either 5×10^9^/ml (undiluted, sample 1) or diluted spores (1∶1, sample 2; 1∶5 sample 3; 1∶10, sample 4; 1∶25 sample 5) from the studied *S. lividans* transformants was dropped onto the agar plates that were subsequently incubated at 30°C for several days.

*Zone of inhibition test*: 10 µl of spores (5×10^9^/ml) were added to 3 ml soft agar and poured onto the respective R2 plates and allowed to solidify. Sterile platelets (6 mm diameter) were added to the bacteria-overlaid plates and saturated with 20 µl of H_2_O_2_ (0.5%), plumbagin (50 mM) or Fe(NH_4_)_2_(SO_4_)_2_ (1 M). Plates were incubated at 30°C before zone of inhibitions were measured after 3 days.

### HbpS protein production, purification and sample preparation

*E. coli* BL21 (DE3) pLysS containing pETHbpS plasmids (Table S1) were used for production of HbpS proteins, their purification was performed using Ni-NTA affinity chromatography, TEV protease cleavage, gel filtration over PD10 columns, dialysis and anion exchange chromatography over a DEAE-sepharose column as previously described [43]. The homogeneity of HbpS protein solutions was analysed by SDS-PAGE and by mass spectrometry. The concentration of purified HbpS solutions was calculated from their absorbance at 280 nm, assuming an ε_280_ of 8250 M^−1^ cm^−1^ (molecular mass = 15 498 Da) and the Bradford assay [49]. For iron-binding studies HbpS proteins were incubated with ammonium iron(II) sulfate [Fe(NH_4_)_2_(SO_4_)_2_] (ratio: 1∶50) in 20 mM Tris/HCl pH 7.0 or 50 mM MOPS (pH 7.0) overnight at 30°C. Other iron ions salts including FeSO_4_, FeCl_2_, FeCl_3_, Fe(ClO_4_)_3_ or FeNH_4_(SO_4_)_2_ were also used as indicated.

### Ferene S staining

Ferene S (3-(2-pyridyl)-5,6-bis(2-(5-furylsulfonic acid))-1,2,4-triazine, disodium salt) can be used as a specific stain to detect iron in proteins [50], [51], [52], [53]. Glassware used with this substance was previously soaked with 1% (v/v) HCl to remove iron ions.

#### Staining in solution

HbpS proteins (each 60 µM) isolated from the corresponding *E. coli* host were incubated with 3 mM Fe(NH_4_)_2_(SO_4_)_2_ as described before. Unbound iron ions were separated from the proteins using gel filtration PD10 columns. For the Ferene S assay 30–80 µl of the reaction mixture were diluted with water to 780 µl. 120 µl 10 M HCl was added and the solution was carefully mixed on a rotator for 10 min. After addition of 100 µl 80% trichloroacetic acid (TCA), the solution was centrifuged at 16000× g for 10 min and the supernatant was transferred into a cuvette. 0.2 ml of 45% acetic acid and 1.8 ml of the freshly prepared Ferene S reagent (containing 45% sodium acetate, 10 mM ascorbic acid and 0.75 mM Ferene S) was added and the sample was immediately mixed. Iron-containing solutions turned blue and the iron content was determined by measuring the absorption at 593 nm. For calibration of iron binding by Ferene S, a standard curve with defined stock solutions of Fe(NH_4_)_2_(SO_4_)_2_ ranging from 0 to 32 nmol were measured. The resulting slope of the calibration line can be used for calculating the iron ions bound by HbpS.

#### Staining in polyacrylamide gels

HbpS proteins (each 20 µM and isolated from the corresponding *E. coli* host) that had been incubated with 1 mM Fe(NH_4_)_2_(SO_4_)_2_ overnight at 30°C were loaded onto a 10% native polyacrylamide gel. After PAGE, the gel was either stained with PageBlue to visualize proteins or subjected to Ferene S staining. Here, the gel was washed with H_2_O for 45 min to remove unbound iron ions and subsequently stained with the Ferene S solution that contains 2% acetic acid, 15 mM thioglycolic acid and 2 mM Ferene S for 2–10 min. The reaction was stopped by washing of the gel with 2% acetic acid.

### Prussian blue staining

The assay was done according to Pulliainen *et al.* [54] with slight modifications. Wild-type and E78A/E81A HbpS proteins (each 20 µM and isolated from the corresponding *E. coli* host) were incubated with 1 mM Fe(NH_4_)_2_(SO_4_)_2_ overnight at 30°C and subsequently loaded onto a 10% native polyacrylamide gel. The equine spleen type I ferritin (Sigma) was used as a positive control and loaded onto a 6% polyacrylamide gel. After electrophoresis the proteins were subjected either for PageBlue staining to visualize proteins or for Prussian blue staining to specifically detect ferric iron. Unbound iron was removed before Prussian blue staining by washing of the gel with a solution containing 50 mM 2-[N-morpholino]ethanosulfonic acid (pH 6.0), 0.15 M NaCl, 5 mM EDTA for 2×15 min at 25°C. The gel was then incubated in a solution of 350 mM HCl, 25 mM potassium ferrocyanide at 25°C for 10 min. The density of the Prussian blue pigment was enhanced by washing of the gel with distilled water (2×5 min) with a subsequent immersion of the gel into freshly prepared solution of 50 mM Tris, 150 mM NaCl (pH 8.0), 10 mM 3,3′-diaminobenzidine tetrahydrochloride, 10 mM H_2_O_2_ and incubated for 5 min in the dark followed by repetitive washing with distilled water.

### Steady-state fluorescence measurements

Tryptophan fluorescence measurements were recorded using a Jasco FP-6500 fluorimeter. 100 µM of HbpS proteins (isolated from the corresponding *E. coli* host) were incubated with 0–10 mM Fe(NH_4_)_2_(SO_4_)_2_ in 50 mM MOPS (pH 7.0) containing 6 mM NH_4_OH at 30°C overnight. NH_4_OH was used as a reducing agent maintaining iron in the ferrous form. Unbound iron ions were removed using PD10 columns. Protein concentrations were estimated again and used for following calculations. Tryptophan was excited with a wavelength of 295 nm. The cell path-length was 1 cm and emission bandwidths were 5 nm. The emission spectrum was recorded from 305 to 550 nm. Each sample was measured three times and the data were averaged to obtain the shown spectra. The dissociation constant was calculated with the equation 1. For exact quantifications, the iron content in the Fe(NH_4_)_2_(SO_4_)_2_ solution was determined by the Ferene S. To minimize the background the buffer was also measured and the spectrum was subtracted from the sample spectra.

Calculation of binding constants (*K_d_*) was done using the equation (Equation 1) listed below as described [55] that takes into account the observed change in fluorescence (ΔF), the maximum of fluorescence change (ΔF_max_), the concentration of HbpS ([E_T_]) and the concentration of the iron ions ([L_T_])
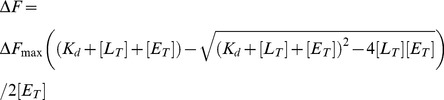
(1)


### Crystallization and structure determination of HbpS-D_78_XXD_81_

HbpS-D_78_XXD_81_ was crystallized at a concentration of 10 mg/ml using the sitting-drop method in a 96-well plate at 295 K. The crystallization condition contained 20% (w/v) polyethylene glycol 3350 and 0.2 M tri-potassium citrate. The crystal was frozen immediately prior to data collection directly in a stream of nitrogen at 100 K without additional cryo-protection. 80 degrees of diffraction data using an oscillation of 0.2 degrees per frame were collected on a Pilatus 6M detector mounted on the EMBL beamline P13 at PETRA III (DESY). The crystal belonged to the space group I422 and contained one molecule in the asymmetric unit. The biological octamer, as previously observed [36], is formed by crystallographic symmetry. The data were processed using XDS [56] and XSCALE [57] to 1.99 Å resolution (Table 1).

**Table 1:** Data collection and refinement statistics of the HbpS-D_78_XXD_81_ crystal structure.

**Data collection**	
Wavelength	1.00
Space group	I422
Cell dimensions	
*a*, *b*, *c* (Å)	77.39, 77.39, 79.86
α, β, γ (°)	90, 90, 90
Resolution (Å)	55-1.99 (2.04-1.99)*
*R*_sym_ (%)	10.3 (86.6)
CC_1/2_ (%)	99.8 (60.9)
<*I*/σ*I*>	11.02 (1.84)
Completeness (%)	99.5 (99.7)
Redundancy	5.6 (5.9)
**Refinement**	
Resolution (Å)	55-1.99 (2.28-1.99)
*R*_work_/*R*_free_ (%)	18.34/23.44 (26.91/33.34)
No. atoms	
Protein	2113
Water	112
*B*-factors (Å^2^)	
Protein	42.7
Water	45.0
R.m.s. deviations	
Bond lengths (Å)	0.004
Bond angles (°)	0.827
Ramachandran plot (%)	
Most favored	98.6
Outliers	0

* Values in parentheses are for the highest-resolution shell.

The structure was solved by molecular replacement using the program Phaser [58] in the Phenix interface [59]. One monomer of the wild-type HbpS (PDB: 3FPV) was used as the search model. Refinement was carried out using the program phenix.refine [60] and model building in Coot [61]. The final model displays good geometry and R/R_free_ factors of 0.183 and 0.234, respectively (Table 1). The structure factors and coordinates have been submitted to the PDB under the code 4BMW.

## Results

### Identification of D/EXXE iron-binding motifs and their location on the HbpS structure

**Figure 1:**
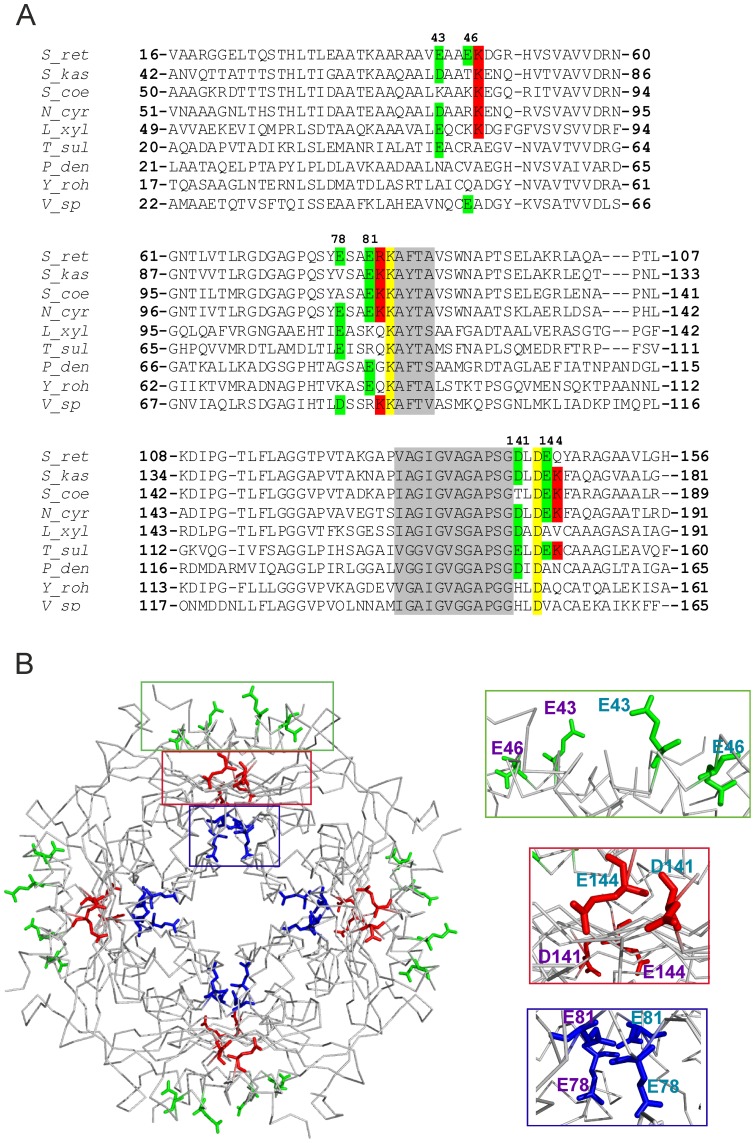
D/EXXE motifs within the HbpS sequence and their position on the octameric structure. (A) Alignments of HbpS from *S. reticuli* (*S_ret*; GI:5834772; numbering according to PDB: 3FPV) and HbpS-like proteins from *S. kasugaensis* (*S_kas*; GI:157059904), *S. coelicolor* A3(2) (S_coe; GI: 8248773) *Nocardia cyriacigeorgica* GUH-2 (*N_cyr*; GI:379707916), *Leifsonia. xyli subsp. xyli str*. CTCB07 (*L_xyl*; GI: 50955378), *Thioalkalivibrio sulfidophilus* HL-EbGr7 (*T_sul*; GI: 220935915), *Paracoccus denitrificans* PD1222 (*P_den*; GI: 119383870), *Yersinia rohdei* ATCC 43380 (*Y_roh*; GI: 238750301) and *Vibrio sp*. MED222 (*V_sp*; GI: 86146209) are shown. Glutamates and aspartates from the studied D/EXXE motifs are marked with green background and their positions on HbpS are indicated. Conserved K83 and D143 are marked with yellow background. Neighbouring and conserved Lys and Arg are in red background. Conserved amino acid regions following E_78_XXE_81_ and preceding D_141_XXE_144_ are in grey background. (B) Positions of the exposed E_43_XXE_46_ (green box) and D_141_XXE_144_ (red box) as well as the internal E_78_XXE_81_ (blue box) on one 4-fold axis of the HbpS octamer are indicated (left). Enlargements of the marked boxes (right) are shown indicating the glutamates/aspartate from one subunit (E43, E46, E78, E81, D141 and E144 written in violet) and the adjacent subunit (Glu and Asp written in turquoise).

By analyses of the iron-mediated formation of dityrosines in HbpS, we identified Tyr-77 as a one of the targets for oxidative attack [38]. This residue is located in the direct vicinity to Glu-78 that together with Glu-81 form an E_78_XXE_81_ motif that might be involved in iron binding and is located in the middle of the HbpS primary structure (Figure 1A). Further sequence analyses led to the identification of two additional motifs, namely E_43_XXE_46_ and D_141_XXE_144_ that are located N-terminally or C-terminally, respectively. Sequence alignments revealed that one E from the central E_78_XXE_81_ and one D/E from the C-terminal D_141_XXE_144_ motifs in HbpS are often present in HbpS-like proteins from other closely and distantly related bacteria (Figure 1A). In contrast, the Glu-43 from the N-terminally located E_43_XXE_46_ motif can be found in only some of the HbpS-like proteins. Analyses of the surrounding regions reveal that a lysine residue (Lys-83 in HbpS) near to the central E_78_XXE_81_ motif and an aspartic acid residue (Asp-143 in HbpS) within the C-terminal D_141_XXE_144_ motif are present in all HbpS-like proteins. Interestingly, in some HbpS-like proteins from distantly related Gram-negative bacteria a histidine residue is located at the corresponding position of Asp-141 from the C-terminal D_141_XXE_144_. Curiously, the region N-terminal to this motif as well as the region C-terminal to E_78_XXE_81_ is very well conserved in all HbpS-like proteins from both Gram-negative and Gram-positive bacteria, suggesting a functional relevance (Figure 1A). Based on the crystal structure of HbpS (PDB: 3FPV), the distribution of the putative iron-binding motifs was analyzed. E_43_XXE_46_ and D_141_XXE_144_ are located on the protein surface; whereas the side chains of the glutamates in E_43_XXE_46_ are orientated to the surrounding solvent, those of the aspartate and glutamate from D_141_XXE_144_ to the inner site. The E_78_XXE_81_ motif is located within the protein core and the side chains of the glutamates are oriented towards each other (Figure 1B).

### Iron-binding properties of generated HbpS mutant proteins

**Figure 2:**
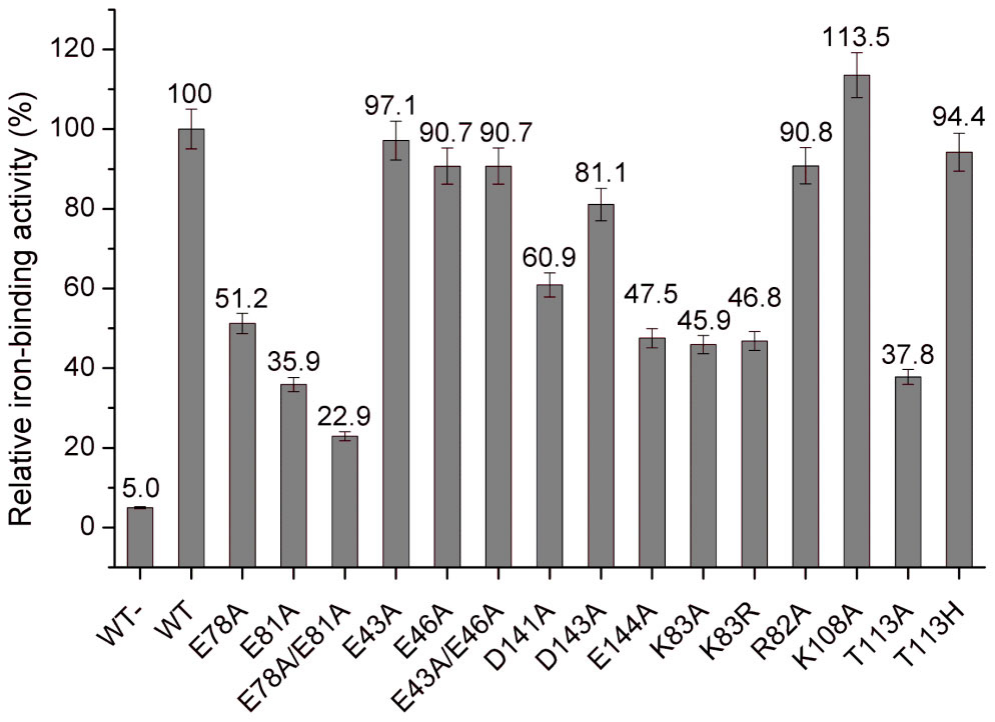
Iron-binding activities of HbpS proteins. The calculated relative iron-binding activities of the wild-type and indicated HbpS mutants regarding the three iron-binding motifs and neighbouring basic residues were measured after treatment with iron ions. The calculated value for the wild-type was set as 100%. The activity of the wild-type sample without previous incubation with iron is also shown (WT-).

In order to analyze the involvement of the D/EXXE motifs and surrounding amino acids in iron binding, corresponding single and/or double *hbpS* mutant genes were generated. The produced proteins were purified to homogeneity from *E. coli* transformants and free of His-tag and iron ions. The wild-type HbpS and the mutant HbpS-K108A [36] were used as controls. The proteins were incubated with iron ions as described in the Material and Methods section. To investigate iron-binding activities quantitatively, the Ferene S staining technique was used. Ferene S is a highly water soluble compound which together with ferrous iron ions forms a stable blue complex that can be measured at 593 nm [50]. Measurements followed by calculations (Figure 2) showed a strong iron-binding activity of the wild type HbpS. This value was set as 100%. Unexpectedly, the mutant K108A showed a high iron-binding activity; Lys-108 was considered to be involved in binding of iron after the HbpS-mediated degradation of haem [36], thus Lys-108 appears to bind exclusively the iron that has been released from haem. Recently, we have elucidated Thr-113 as a haem-binding site in HbpS [35]. Iron-binding assays using T113H (with strong haem-binding activity) and T113A (with reduced haem-binding activity) mutant proteins free of haem revealed that T113H binds iron in almost identical manner like the wild-type, the T113A mutant, however, showed reduced iron-binding activity (to 37%) (Figure 2). We conclude that under the reaction conditions assayed the calculated value of 100% for the wild-type includes the binding of iron at Thr-113.

Mutations of the glutamates from the surface exposed motif E_43_XXE_46_ led to only minor decrease in iron binding. The single mutants E43A and E46A as well as the double mutant E43A/E46A retained 97.1%, 90.7% and 90.7% iron-binding activity, respectively. Exchanges of the aspartic or glutamic acid from another surface exposed motif D_141_XXE_144_ resulted in higher levels of decrease in iron binding, namely 60.9% for D141A and 47.5% for E144A. In contrast to these single mutants, a generated double mutant protein D141A/E144A was not stable after purification, thus iron-binding assays were not conducted. The highest decrease (up to 22.95%) of iron-binding activity was recorded for the double mutant E78A/E81A of the EXXE motif located in the core of HbpS. The single mutants E78A and E81A showed also strong decrease (51.2% and 35.9%, respectively).

The above described sequence analyses showed the conservation of Lys-83 and Asp-143 within different HbpS-like proteins; these amino acid residues are additionally located near or within the described E_78_XXE_81_ and D_141_XXE_144_ motifs, respectively. Thus, corresponding mutant proteins were also produced, purified and analysed (Figure 2). Interestingly, the mutant protein HbpS-K83A showed a strong decrease of iron binding (to 45.9%); the replacement of lysine by another basic amino acid residue, K83R, showed almost the same effect (to 46.8%). We generated also the mutant HbpS-R82A (R82 is not well conserved) that showed only a low decrease (to 90.8%). Analysis of iron binding by HbpS-D143A showed a decreased to 81.1% that in comparison to D141A (60.9%) and E144A (47.5%) is not that high; it indicates, however, that Asp-143 plays a role in iron binding by D_141_XXE_144_.

Summarizing, these data show that E_78_XXE_81_ together with K83 in the core of HbpS possess the strongest iron-binding activity followed by D_141_XXE_144_. The N-terminally located E_43_XXE_46_ participates only weakly in the binding of iron.

### HbpS binds Fe(II) and oxidizes it to Fe(III)

**Figure 3:**
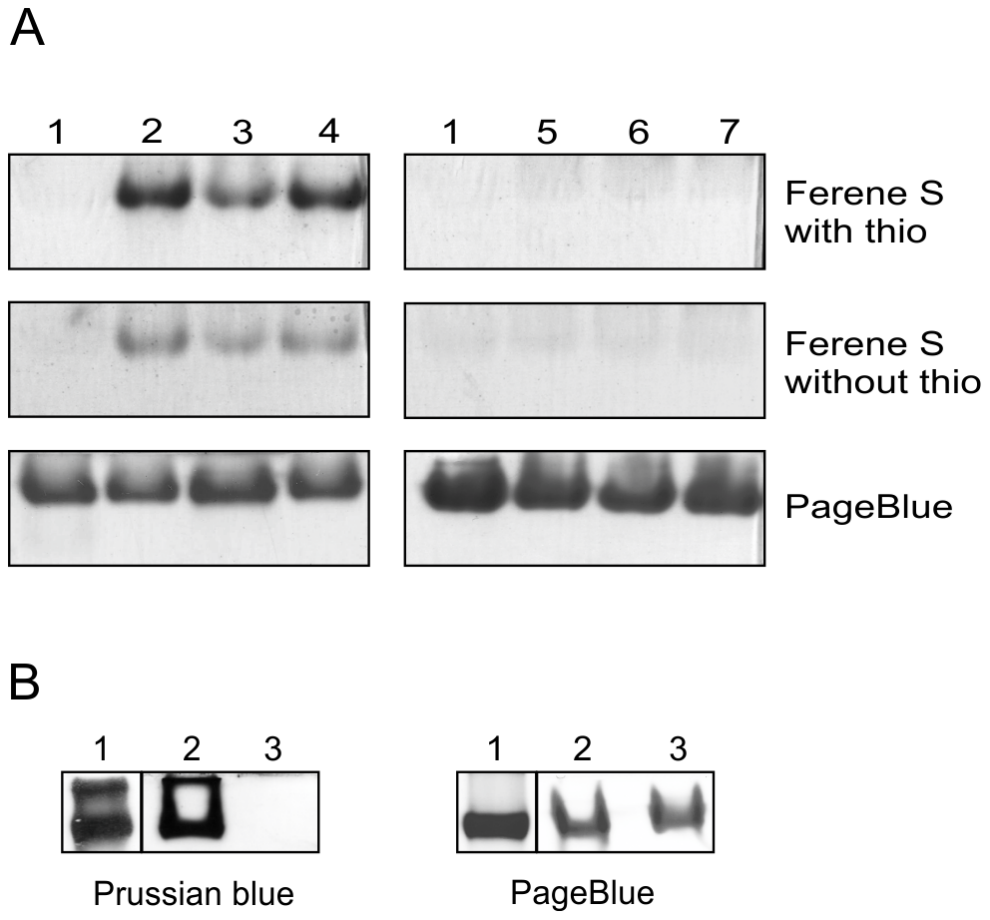
HbpS binds Fe(II) and oxidizes it to Fe(III). (A) Aliquots of wild-type HbpS without previous incubation with iron ions (lane 1) and incubated with either the ferrous iron salts Fe(NH_4_)_2_(SO_4_)_2_ (lane 2), FeCl_2_ (lane 3) and FeSO_4_ (lane 4) or with the ferric iron salts FeNH_4_(SO_4_)_2_ (lane 5), FeCl_3_ (lane 6) and Fe(ClO_4_)_3_ (lane 7) were loaded onto native PAA gels (3x). Ferene S staining was performed with (top) or without thioglycolic acid (thio) (middle). As a control proteins were stained with PageBlue (bottom). (B) Wild-type HbpS (lane 2) and the mutant HbpS protein E78A/E81A (lane 3) were incubated with Fe(NH_4_)_2_(SO_4_)_2_. Reaction mixtures were split into two parts and proteins were subjected to native PAGE. PageBlue was used to stain proteins (right) and Prussian blue staining to specifically detect Fe(III). Equine spleen type ferritin (lane 1) was used as a positive control for Prussian blue staining.

Due to their slight different electrochemical properties, ferrous (with incompletely filled orbital d^6^) and ferric (with incompletely filled orbital d^5^) iron ions might have different preferences for certain protein ligands and *vice versa* [4]. To investigate the iron ion form interacting with HbpS, Ferene S analyses on native polyacrylamide gels were performed. It is important to note here that Ferene S complexes Fe^2+^ ions, but not Fe^3+^. The reducing agent thioglycolic acid used during Ferene S staining either maintains or induces the reduced state of the coordinated iron ion [62]. Wild-type HbpS (20 µM) was pre-incubated either with the ferrous iron salts FeCl_2_, FeSO_4_ and Fe(NH_4_)_2_(SO_4_)_2_ or the ferric ones FeCl_3_, Fe(ClO_4_)_3_ and FeNH_4_(SO_4_)_2_ in 50 mM MOPS (pH 7.0) before loading onto the gel. After electrophoresis, the gel was stained with Ferene S solution that either contains or lacks the reducing agent thioglycolic acid. In both cases a positive Ferene S staining was achieved for the wild-type that had been previously incubated with ferrous iron salts (Figure 3A; lanes 3–5). In contrast, no staining was observed for HbpS incubated with ferric iron salts (Figure 3A; lanes 6–8), indicating that HbpS specifically binds ferrous iron (Figure 3A).

EXXE motifs within ferritins, bacterioferritins and Dps proteins are involved in binding of ferrous iron and in their oxidation *via* the so-called ferroxidase reaction [16], [17]. The Prussian blue staining is a suitable method to specifically detect Fe(III) in proteins and was used by others to analyze ferritins or Dps proteins [54], [63]. Wild-type HbpS and the double mutant E78A/E81A were pre-incubated with the ferrous iron salt Fe(NH_4_)_2_(SO_4_)_2_ before loading onto a native polyacrylamide gel. After electrophoresis the proteins were stained with Prussian blue, as described in the Material and Methods section. The positive control (ferritin) as well as the wild-type were stained, indicating that HbpS had oxidized Fe(II) to Fe(III) (Figure 3B).

### HbpS requires its oligomeric assembly for iron binding

**Figure 4:**
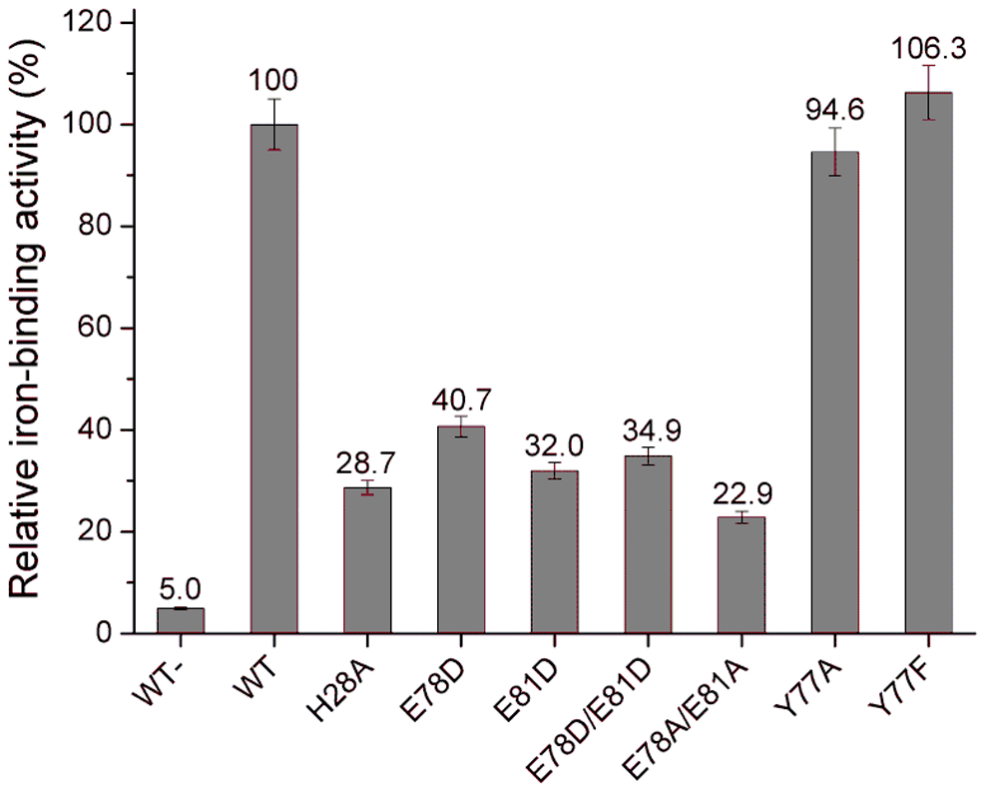
Iron-binding assays using monomeric HbpS and furter mutants with exchanged E by D or A (within the internal E_78_XXE_81_ motif) or Y by A or F (from the adjacent Y77). Relative iron-binding activities of the wild-type as well as of the mutants H28A (building only monomers), E78D, E81D, E78D/E81D, E78A/E81A, Y77A and Y77F previously incubated with iron ions are shown. The activity of the wild-type sample without previous incubation with iron is also shown (WT-).

Because each of the D/EXXE motifs in HbpS is oriented to each other in the interface of adjacent subunits, we hypothesized that the oligomeric assembly in HbpS may play a crucial role during iron binding. In fact, when binding experiments were performed using an HbpS-H28A mutant protein that forms only monomers [36], but retains all D/EXXE motifs unchanged, a strongly reduced iron-binding activity was recorded (up to 28.7%) (Figure 4). These data implicate that the distance between the side chains of corresponding iron-binding amino acids located in the interface between two adjacent subunits could be important. To address this point and taking into account that the E_78_XXE_81_ motif shows the strongest iron binding, E78D, E81D as well as E78D/E81D mutants were generated. One could expect that the exchange of E to D would not have any significance relevance for iron- binding behaviour, but analyses of isolated mutant proteins incubated with ferrous iron revealed that all three mutants show strong decreased iron-binding activity, namely E78D up to 40.7%, E81D 32% and E78D/E81D 34.9% (Figure 4). In common with glutamates, aspartates (at pH 7.5) have a negative charge and can participate in ionic interactions. However, they are shorter by one CH_2_ group than glutamates. This feature might explain why the Asp mutants do not bind iron well.

### Analysis of the crystal structure of HbpS-D_78_XXD_81_

**Figure 5:**
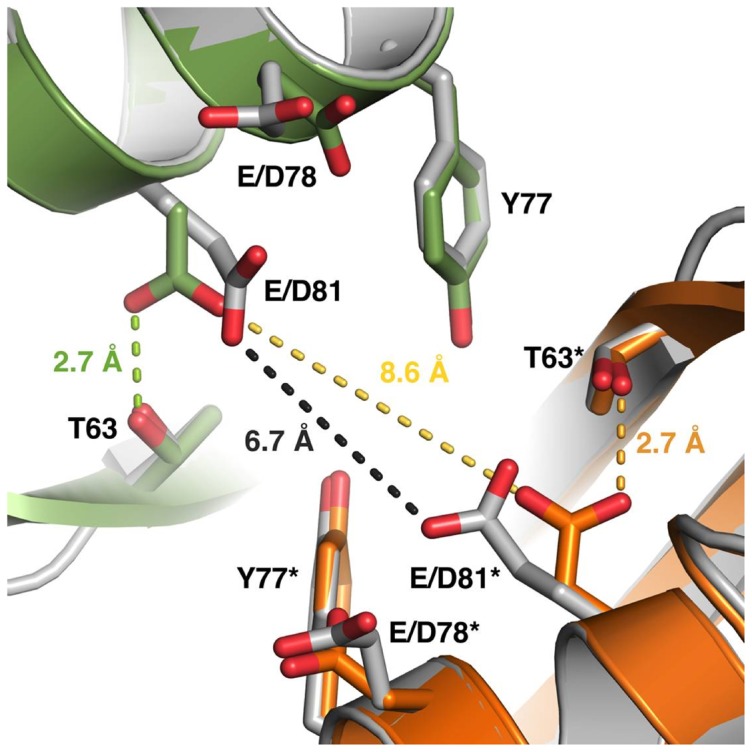
Structural rearrangements due to the E78D/E81D mutations. The side chains of the E/D78 and E/D81 residues in the wild-type and the mutated HbpS point towards the corresponding side chains from a neighboring monomer in the octameric assembly. The distance of the two E81 side chains in the wild-type protein (gray) is 6.7 Å, which would allow for iron coordination. Other nearby residues include Y77 and T63. In the mutated protein, this distance has increased to 8.6 Å. Furthermore, the side chain of D81 has turned to form a hydrogen bond with T63. The two monomers in the mutant HbpS are colored green and orange. The residues discussed are labeled, and the asterisk denotes the residues from the symmetry-related molecule in the crystal. The distances between the corresponding E/D81 residues as well as the hydrogen bond between D81 and T63 are marked with dotted lines.

In order to gain structural insight into the effects of the E78D and E81D mutations on the iron binding of HbpS, the HbpS-D_78_XXD_81_ mutant protein was purified and crystallized. As expected, the mutations induce no major structural rearrangements and the mutant protein has retained the octameric assembly seen for the wild-type HbpS (PDB: 3FPV). In the wild-type protein, the glutamates (Glu-78 and Glu-81) point towards the central cavity of the octamer and are apposed by the corresponding residues from a second monomer, thus creating a cluster of four negatively charged residues. In addition, Tyr-77 from two subunits is present in the same cluster (Figure 5); iron-binding assays using HbpS-Y77F and HbpS-Y77A mutant proteins revealed that this amino acid is not involved in iron binding (Figure 4). In the wild-type protein, the distance between the Glu-81 residues from two apposing subunits is 6.7 Å, whereas the distance of the corresponding aspartate residues in the mutant protein is 8.6 Å (Figure 5). Furthermore, in the mutant protein, Asp-81 is engaged in a hydrogen bond with Thr-63, which can be expected to further decrease the ability of this side chain to take part in metal coordination.

### Iron-mediated quenching of tryptophan fluorescence on HbpS proteins

**Figure 6:**
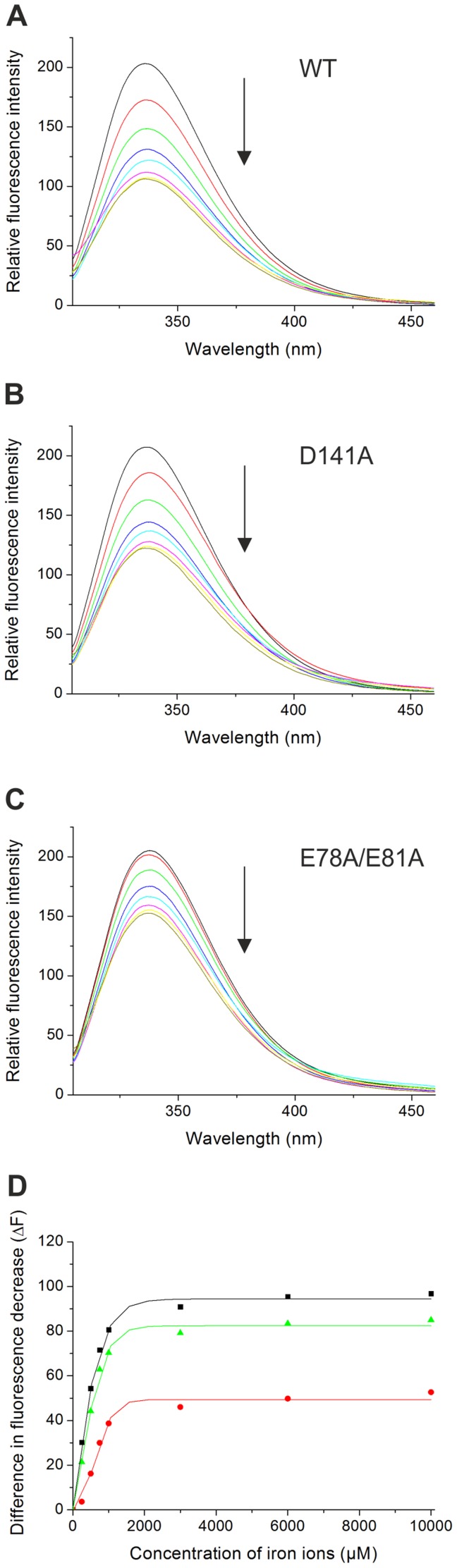
Iron-based quenching of Trp fluorescence of HbpS proteins. (A–C) HbpS proteins were treated with increasing concentrations (up to 10 mM; marked by the arrow) of ferrous iron ions, as indicated in the Material and Methods section. The fluorescence spectra of the wild-type (WT) as well as of the mutants D141A and E78A/E81A were subsequently measured. (D) Differences in fluorescence (ΔF) were plotted against the concentrations of titrated ferrous iron for HbpS WT (▪), D141A (▴) and HbpS E78A/E81A (•).

To determine the binding affinity and the stoichiometry of the interaction between iron and HbpS, quenching of the tryptophan (Trp) fluorescence in iron-dependent manner was monitored. It is known that intrinsic tryptophan fluorescence emission is influenced by interacting iron ions in iron-binding proteins [55], [64], [65]. This quenching arises from the Förster energy transfer (FRET) of the tryptophan to absorption bands generated by iron binding to the protein [64]. HbpS has only one tryptophan (Trp-90) that is located relatively close to the inner (E_78_XXE_81_; ∼12 Å) or to the surface-exposed (D_141_XXE_144_; ∼10 Å) iron-binding motif within the octameric HbpS. Trp fluorescence spectra from HbpS proteins (each 27 µM) previously incubated with different concentrations of Fe(NH_4_)_2_(SO_4_)_2_ were recorded (Figure 6). With increasing concentrations of iron, the quenching of Trp fluorescence from the wild-type HbpS increased continuously until saturation at 2500 µM Fe(NH_4_)_2_(SO_4_)_2_ was achieved (Figure 6A, D). Saturation for the mutants HbpS-D141A (Figure 6B, D) and HbpS-E78A/E81A (Figure 6C, D) was achieved already at concentrations of 1800 µM or 1400 µM Fe(NH_4_)_2_(SO_4_)_2_, respectively. Based on the spectra, ΔF values were calculated (Figure 6D) and used to quantify *K_d_* (according to Equation 1). The calculated binding affinity (15×10^−6 ^M) for the wild-type HbpS was ∼8 times higher than that one (115×10^−6^ M) from the HbpS-E78A/E81A mutant and ∼2 times higher (34×10^−6 ^M) than that of the HbpS-D141A mutant. A detailed analysis using additional concentrations of iron ions was done for the wild-type HbpS (Figure S2); this confirmed the *K_d_* determined above.

These diminished quenching values and *K_d_* for the HbpS mutant proteins correlate with the Ferene S data showing a moderate or respectively strong reduced iron-binding activity for both mutant proteins (Figure 2).

### HbpS supports protection of streptomycetes from hazardous effects of iron-mediated oxidative stress through its E_78_XXE_81_ signature

The data presented above revealed that the E_78_XXE_81_ motif within the octameric HbpS is the main binding site for Fe^2+^ iron. Based on the saturating concentrations of iron ions inducing Trp fluorescence quenching (Figures 6D and S2), the octameric wild-type HbpS binds up to 100 iron ions. We assumed therefore that because of this iron-sequestering ability, HbpS alone provides protection against iron-based oxidative stress *in vivo*. To investigate this hypothesis, we generated a number of plasmid constructs that can be used to transform protoplasts from streptomycetes and allow the constitutive expression of the wild-type and mutant *hbpS* genes. For cloning, we used the pWHM3 plasmid that replicates in *E. coli* as well as in streptomycetes [66] and the pUC18 derivate plasmid construct pUKS13 [45] that contains the *furS-cpeB* operon with an inactivated *furS* that is not able to repress the transcription of *furS-cpeB*; at these conditions the strong promoter in front of *furS* is constitutively active. Using a multi-step cloning strategy (see Material and Methods section), the *cpeB* coding sequence on pUKS13 was replaced by either the wild type *hbpS* or different *hbpS* mutant genes (encoding E78A, E81A or E78A/E81A). All cloned genes contained their original sequence encoding the signal peptide to ensure Tat-dependent secretion. The plasmid constructs obtained pWHbpS, pWHbpS-E78A, pWHbpS-E81A and pWHbpS-E78A/E81A (Table 1) were used to transform *S. lividans* protoplasts. The production of extracellular HbpS proteins in the transformants obtained was confirmed by Western blot using *anti*-HbpS antibodies (Figure S1). Their sensitivity against the redox-cycling compounds H_2_O_2_ and plumbagin as well as high concentrations of iron ions and haemin (the oxidized form of haem) was studied by three different procedures according to their applicability.

**Figure 7:**
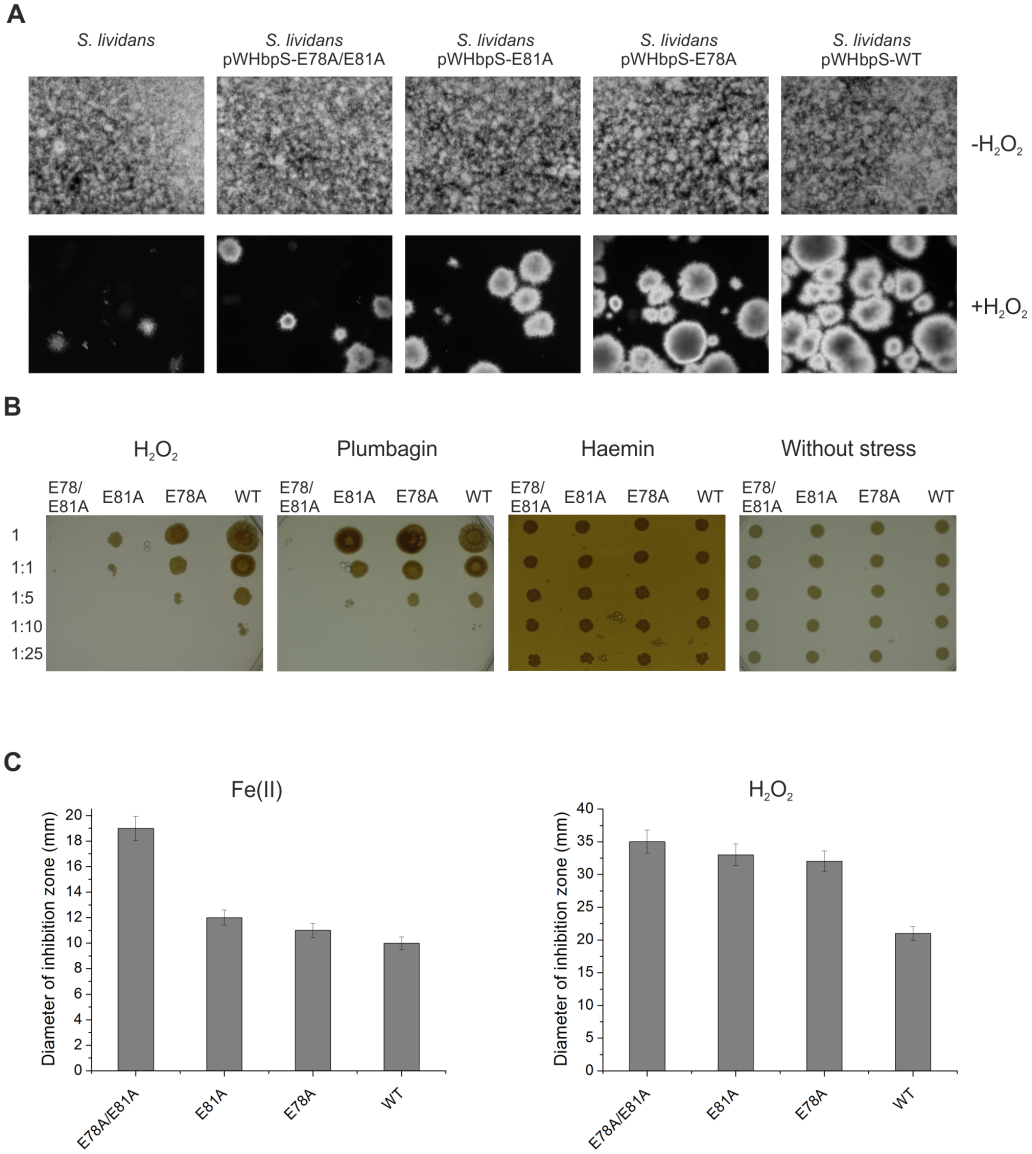
Growth of *S. lividans* transformants under different oxidative-stressing conditions. Identical number of spores of the host strain *S. lividans* and its transformants containing either pWHbpS-WT or pWHbpS-E78A or pWHbpS-E81A or pWHbpS-E78A/E81A were inoculated in cultivation media (see Material and Methods section) lacking or containing the indicated stressors (H_2_O_2_, plumbagin, haemin or ferrous iron ions). Their growth was analysed microscopically (A), on agar plates (B) and with zone of inhibition assays (C).

At first the growth of *S. lividans* transformants and the host strain *S. lividans* in microtiter plates was monitored microscopically in media either lacking or containing 0.0025% (v/v) H_2_O_2_ (Figure 7A). In the absence of the stressor all four transformants as well as the host strain grew identically with mycelia evenly distributed over the whole microtiter cell (Figure 7A, top). In presence of peroxide the growth of the host strain as well as of the transformants containing pWHbpS-E78A, pWHbpS-E81A or pWHbpS-E78A/E81A was considerably slower compared to that one from the transformant harbouring pWHbpS (Figure 7A, bottom). Moreover, the hyphae of the host strain and transformants with mutated *hbpS* genes agglomerated faster during growth. Remarkably, the host strain and the *S. lividans* pWHbpS-E78A/E81A transformant showed the poorest growth, followed by *S. lividans* pWHbpS-E81A and *S. lividans* pWHbpS-E78A. Growth of strains using iron ions, haemin or plumbagin could not be monitored, as iron ions and haemin precipitated rapidly in the liquid medium, and plumbagin was extremely toxic (even at a concentration of 0.0075 mM), inhibiting completely the growth of all investigated strains (not shown).

With the second procedure, the growth of the studied *S. lividans* transformants was followed on agar plates without or with H_2_O_2_ (0.0025% (v/v)) or with plumbagin (0.005 mM) or with haemin (100 µM). In this experiment, aliquots (1 µl) of undiluted (sample 1) and different dilutions (samples 1∶1, 1∶5, 1∶10 and 1∶25) of spores were dropped on the corresponding plate and their growth was documented daily. On plates without any stressor a clear growth of all four strains and in all samples was observed already after one day of incubation (Figure 7B, right). On plates containing 0.0025% (v/v) H_2_O_2_, growing mycelia were visible only for the *S. lividans* pWHbpS transformant within the sample 1 after one day of incubation (not shown). After eight days, the growth of *S. lividans* pWHbpS was detected up to the sample 1∶10, by *S. lividans* pWHbpS-E78A up to the sample 1∶5, by *S. lividans* pWHbpS- E81A up to the sample 1∶1 and for *S. lividans* pWHbpS-E78A/E81A a minimal growth was detected only within the sample 1 (Figure 7B, left). Additional incubations on plates containing 0.005 mM plumbagin yielded similar results (Figure 7B, middle) with the best growth for *S. lividans* pWHbpS and the poorest one for *S. lividans* pWHbpS-E78A/E81A. In the presence of haemin all strains tested displayed an identical haemin-resistant phenotype (Figure 7B, middle). Again, iron ions could not be tested as these tended to precipitate rapidly in the used medium.

The third procedure consisted of zone of growth inhibition assays. Here, a high concentration of iron ions (20 µl of 1 M solution) could be used, as any precipitation during dropping on the papers discs was observed. After three days of incubation, the broadest zone (19 mm) of growth inhibition was recorded on the plate containing *S. lividans* pWHbpS-E78A/E81A, followed by *S. lividans* pWHbpS-E81A (12 mm), *S. lividans* pWHbpS-E78A (11 mm) and *S. lividans* pWHbpS (10 mm) (Figure 7C, left). Additional assay on plates containing 0.5% (v/v) H_2_O_2_ (Figure 7C, right) yielded similar results with the broadest zone of growth inhibition for *S. lividans* pWHbpS-E78A/E81A and the slightest one for *S. lividans* pWHbpS. In the presence of haemin no zone of growth inhibition was observed for each of the tested transformants (data not shown).

Notably, the overall results of these three procedures (each done in triplicate) showed that while the *S. lividans* transformant pWHbpS-E78A/E81A displayed the highest sensitivity against all tested stressors, followed by *S. lividans* pWHbpS-E81A and *S. lividans* pWHbpS-E78A, the highest resistance was observed for *S. lividans* pWHbpS harbouring the wild-type *hbpS* gene. These data are in complete agreement with the biochemical data showing that in comparison to the wild-type with the highest activity (set as 100%), HbpS-E78A/E81A displayed the lowest iron-binding activity (22.9%), followed by HbpS-E81A (35.9%), and HbpS-E78A (51.2%) (Figure 2).

## Discussion

In this work, we have biochemically characterized the iron-binding sites within the octameric protein HbpS and provided evidences as to their physiological relevance for *Streptomyces*. Previous data and analyses of the 3D crystal structure of the octameric HbpS led to the identification of three iron-binding motifs D/EXXE, two (E_43_XXE_46_ and D_141_XXE_144_) are located on the surface and one (E_78_XXE_81_) in the core of the octamer. Although all these motifs are not well conserved in HbpS-like proteins from closely and distantly related bacteria, there are single glutamates or aspartates showing variable degrees of conservation within the corresponding regions. In addition, we found a high degree of conservation within the amino acid regions adjacent the C-terminal (D_141_XXE_144_) and the central (E_78_XXE_81_) motif (Figure 1), suggesting an important functional role. Iron-binding assays revealed that one of the exposed motifs (E_43_XXE_46_) in HbpS is only weakly involved in iron binding, whereas the second (D_141_XXE_144_) has a higher iron-binding activity. The internal E_78_XXE_81_, however, shows the tightest binding. It is worth pointing out that also the single mutant proteins E78A, E81A, D141A or E144A variably retained (51.2%, 35.9%, 60.9% and 47.5%, respectively) iron-binding activity, suggesting that on the one hand the contribution of each Glu/Asp during the process of iron binding is important, and on the other hand their contribution is distinct. The Ftr1p iron-binding protein from *Saccharomyces cerevisiae* is the permease component of the Fet3p-Ftr1p iron-uptake complex and includes several D/EXXE motifs. Studies have shown that single elements (D or E) of an iron-binding motif contribute variably during the process of iron binding [26]. Interestingly, the iron permease CaFtr1P from *Candida albicans* contains five EXXE motifs, of which only one is essential for iron transport. N-terminally to the first E of this motif a positively charged amino acid (Arg or Lys) is present that has been shown to be also essential for iron transport [67], [68]. In ferritins and Dps proteins a positively charged amino acid is located adjacent to the second E of the corresponding iron-interacting motif [29], [68]. The exact role of the Arg or Lys is not clear; it is expected that these basic amino acids stabilize the structure of the region of the protein that interacts with iron. Notably, the E_78_XXE_81_ motif in HbpS that displays the highest iron-binding activity is followed by Arg (R82) and Lys (K83). Whereas the mutant protein HbpS-R82A retained almost a complete iron-binding activity (90%), the K83A mutant showed a strong decrease in iron binding (45.9%). Thus, flanking basic amino acids to EXXE motifs seem to be determining features for proteins using this motif for iron binding. Additionally, the overall octameric assembly in HbpS is crucial for iron-binding activity, as mutant HbpS proteins forming only monomers lack this activity. Moreover, comparative analyses using the high resolution (1.99 Å) crystal structure of HbpS-E78D/E81D and of the wild-type HbpS revealed that the proper orientation and proximity of the side chains of the glutamates from neighbouring E_78_XXE_81_ motifs within the octamer are also essential for iron binding. In HbpS-E78D/E81D, the side chains of the aspartates are shortened by one CH_2_ group, and it can be assumed that this weakens the coordination of an iron. In fact, the iron-binding activity of this mutant is considerably weaker than that from the wild-type (Fig. 4). The overall octameric assembly of HbpS-D_78_XXD_81_ remains unchanged and no major structural rearrangements were detected. Thus, not only the presence of an EXXE motif within the amino acid sequence is important, but the structural arrangement of iron-coordinating amino acids within the overall protein structure is critical.

**Figure 8:**
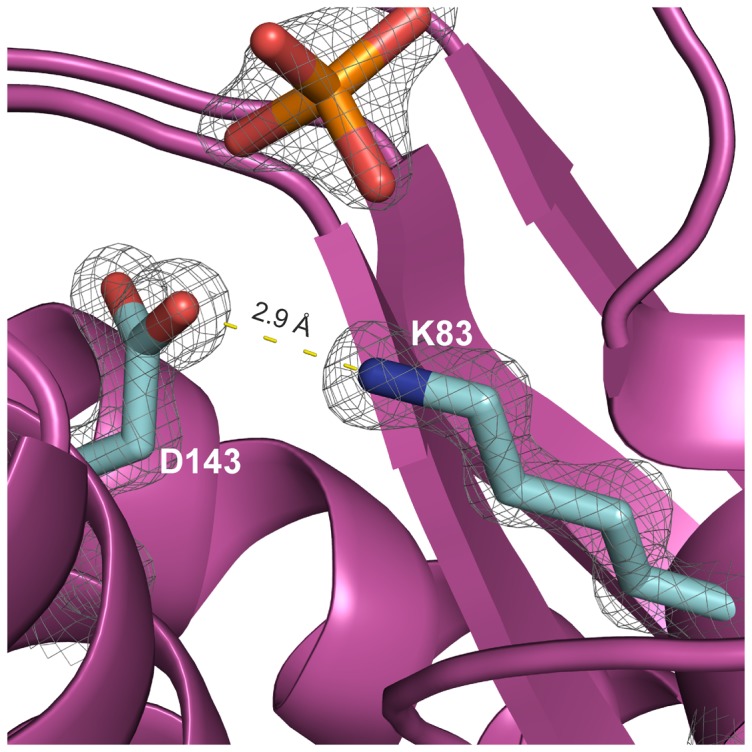
An illustration showing interacting Lys-83 (K83) and Asp-143 (D143) within one HbpS subunit (map contoured at 1.5 sigma). The indicated distance between them is marked with dotted lines. A phosphate group (in orange) possibly stabilizing the interaction is also shown.

**Figure 9:**
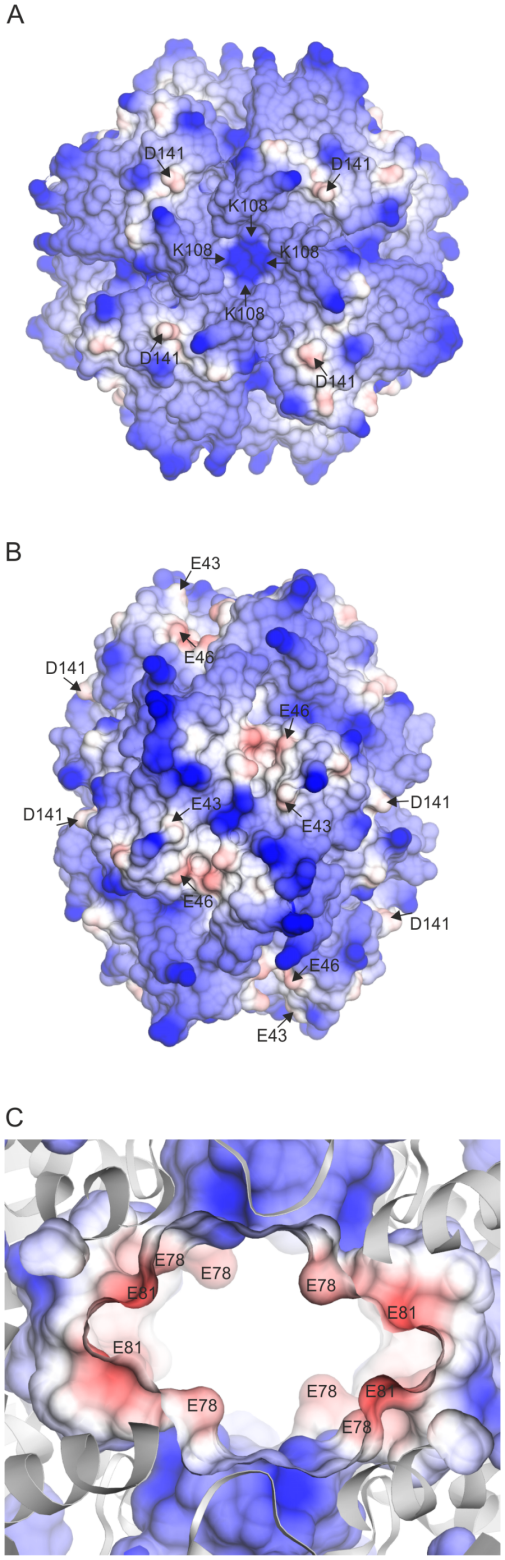
Electrostatic surfaces of the octameric HbpS. (A) and (B) show some of the exposed E43, E46 and D141 (red) residues within the predominantly positive charged (blue) HbpS surface. K108 as well as E43, E46 and D141 are indicated by arrows. (C) An enlargement of the octameric structure shows the roughly spherical and predominantly negative charged (red) core in which the indicated E78 and E81 from different subunits are shown.

Sequence comparisons revealed that Lys-83 as well as Asp-143 are completely conserved in all HbpS-like proteins from both Gram-positive and Gram-negative bacteria (Figure 1). Asp-143 is located within the D_141_XXE_144_ motif and also plays a role in binding iron, as the D143A mutant shows a diminished iron-binding activity. Based on the crystal structure of the wild-type HbpS, we identified physical contacts between the side chains of both Lys-83 and Asp-143 that are separated only by 2.9 Å and likely form salt bridges (Figure 8). These data strongly suggest that both amino acids connect the surface-located D_141_XXE_144_ with the E_78_XXE_81_ motif that is located in the core of the octameric HbpS. We propose therefore that iron ions are transported from the outside to the inside of HbpS using a highly specific route. Based on Figure 1 showing the position of the D/EXXE motifs within the HbpS octamer, it is likely that the specific route includes the initial binding of iron by the exposed E_43_XXE_46_ and D_141_XXE_144_ motifs that support the transport of iron into core of HbpS. This is in accordance with the biochemical data showing a higher binding affinity for E_78_XXE_81_, implying that the exposed motifs are primarily involved in recognition and guidance of iron to the roughly spherical core of the octameric HbpS. Analyses of the electrostatic surface of HbpS additionally revealed that the surface is predominantly positive charged with some negatively charged regions which include E_43_XXE_46_ and D_141_XXE_144_ (Figure 9A, B). The positively charged amino acids on the octameric surface include K108 (Figure 9A) that binds iron released after the HbpS-mediated degradation of haem [35]. A view in the inside of this surface shows negative charged platforms distributed around the surface of the core, which are formed principally by adjacently ordered E_78_XXE_81_ motifs from different subunits within the octameric assembly (Figure 9C) that as mentioned above is essential for the interaction with iron. We assume, therefore, that the D/EXXE motifs studied in HbpS mediate the nucleation of iron ions. Such a process of nucleation is one feature of ferritins and Dps proteins, their assemblies reveal a hollow spherical structure comprising 24 or 12 subunits, respectively [14], [29]. In Dps from *Deinococcus radiodurans* and the plant ferritin SFER4 the internal negative charge of the sphere is formed by different Glu within an EXXE motif; these acidic residues within these platforms are suggested to mediate the iron nucleation that includes the oxidation of Fe(II) to Fe(III) [29], [67], [70]. In the deinococcal Dps these glutamates have close contacts with Lys and Arg that together might form salt bridges and be relevant for the nucleation events [29], [69]. While, HbpS does not show any substantial sequence similarities with Dps or ferritins, its overall octameric spherical assembly is comparable. It is composed of eight identical subunits with an internal cavity of oblate cross sections of 22 and 32 Å. Surprisingly, HbpS in analogy to ferritins and Dps proteins is able to oxidize Fe(II) to Fe(III), suggesting that sequestered iron ions remain stored in the core of HbpS. Moreover, based on the fluorescence spectroscopic data (Figures 6D and S2), the octameric HbpS was deduced to bind up to 100 iron ions. This value is obviously lower than that one characterized for Dps (up to 500) and ferritins (up to 4500); it shows, however, that HbpS functions as an iron-sequestering biomolecule.

Sequestration of iron ions is one of the main protection systems allowing bacteria and other organisms to counteract the hazardous effects of the iron-based Fenton reaction. Our comparative studies demonstrate that the growth of *Streptomyces* strains secreting HbpS mutant proteins with substituted glutamates within the iron-binding motif E_78_XXE_81_ is strongly diminished during cultivation under oxidative stress conditions (i.e. high concentrations of iron ions, hydrogen peroxide or plumbagin). Notably, strains producing HbpS proteins that contain single substitutions (either E78A or E81A) are less sensitive to the stressors than that one producing HbpS-E78A/E81A. These observations are in agreement with biochemical data showing the lowest iron-binding activity for the double mutant. Our interpretation is that the secreted double mutant is not able to sequester ferrous iron from the surrounding environment. Consequently, unbound iron reacts with hydrogen peroxide, leading to the generation of hydroxyl radicals and other reactive oxygen species that mediate the oxidative attack of macromolecules within *Streptomyces* mycelia.

Recently, we have identified a haem-binding site in HbpS, at Thr-113 and additionally shown that HbpS degrades haem [35], [36]. The iron released is expected to be captured by Lys-108 on the surface of HbpS and support the activation by autophosphorylation of the two-component sensor kinase SenS under oxidative stressing conditions. Intriguingly, iron-binding assays using HbpS-K108A showed that this mutant binds irons in a similar manner like the wild-type protein (Fig. 2), indicating that under the employed incubation conditions Lys-108 is not involved in iron binding. Analysis of the octameric structure shows that Thr-113 is located closer to Lys-108 than to the main iron-binding site Glu-78/81 (Figure S3), supporting our hypothesis that the source of iron to be bound at Lys-108 is exclusively the degraded haem. Further experiments revealed that Glu-78/81 are not involved in haem binding (Figure S4); this is in agreement with the presented physiological data showing that the corresponding E78A, E81A or E78A/E81A mutants display identical levels of resistance against high concentrations of haem compared to that one from the wild-type (Figure 7). In this context, it is noteworthy to mention that the oligomeric assembly of HbpS is crucial for iron binding (Fig. 4) but not for binding of haem [36].

Beside the high capacity to store iron, some bacterial ferritins, the so-called bacterioferritins, additionally interact with haem. For instance, the 24-mer bacterioferritin from *E. coli* binds 12 haem molecules [71]. Studies have shown that haem is not involved in the oxidation of Fe(II) to Fe(III) [72]. Over the last years it was unclear which role is played by haem in ferritins in dealing with iron. Recently, it was demonstrated that haem is involved in the release of iron from the bacterioferritin core by electron transfer processes [21]. The recycling of iron in other ferritins has been related to the proteolytic degradation of the macromolecule or the presence of high affinity iron chelators [21], [73]. Interestingly, our previous data showed that HbpS undergoes proteolytic degradation after long exposure to oxidative stress [38], implying that this might be a pathway for release of the internalized iron. In future, studies will be done in order to clarify whether haem plays any role in the nucleation or release of iron from HbpS.

## Conclusions

Our previous reported works have characterized HbpS as the accessory and sensory component of the two-component system SenS-SenR that together mediate a genetic response of *Streptomyces* against iron- and haem-based oxidative stress. Moreover, studies have shown that HbpS is a novel type of haem-binding protein that degrades haem *in vitro* and *in vivo*. The HbpS-mediated turnover of haem provides the bacterium with an additional defence mechanism on the presence of hazardous concentrations of haem. In this work, we have shown that this multifunctional extracellular octameric protein binds ferrous iron through D/EXXE signatures. Analyses of the 3D structures and the *in vitro* and *vivo* data obtained led to the assumption that these motifs are involved in the transport of high quantities of iron ions into the core of the HbpS octamer. With this sequestration function HbpS complexes ferrous iron from the surrounding environment and oxidizes it to its ferric from, making them inaccessible to react with hydrogen peroxide *via* the Fenton reaction, the products of which are highly reactive and are damaging to a number of different macromolecules. The sequestration and oxidation of high quantities of ferrous iron is a protective mechanism that has been elucidated for ferritins, bacterioferritins and Dps proteins. These proteins also contain EXXE motifs that play a role in iron-nucleation events. Since there is not a clear structural similarity between HbpS and these proteins, a common functional iron-sequestering and iron-oxidizing role can be suggested. An important difference is, however, the extracellular location of HbpS, and hence its role in the defence against environmental iron ions and in the initial protection of *Streptomyces* macromolecules located near to the extracellular space. In future, it will be challenging to analyze to what extent HbpS protects cell wall and membrane compartments from the attack of iron-mediated reactive oxygen species. Because HbpS-like proteins are widespread along a high number of Gram-positive and Gram-negative bacteria, the presented data as well as future works will be relevant for different organisms.

## Supporting Information

Figure S1 HbpS proteins are secreted in studied *S. lividans* tranformants. Culture filtrates of *S. lividans* transformants containing either pWHbpS-WT (lane 2) or pWHbpS-E78A (lane 3) or pWHbpS-E81A (lane 4) or pWHbpS-E78A/E81A (lane 5) or pWHM3 (lacking *hbpS*; lane 1) were subjected to Western blot analysis using *anti*-HbpS antibodies.
(TIF)

Figure S2 Iron-based Trp Fluorescence quenching of HbpS. (A) Fluorescence spectra of HbpS previously incubated with increasing concentrations (up to 10 mM; marked by the arrow) of ferrous iron ions. B) Differences in fluorescence (ΔF) were plotted against the concentration of titrated ferrous iron.
(TIF)

Figure S3 Arrangement of the haem-binding and some of the iron-binding sites within HbpS. The distance (in Å) from the haem-binding site at T113 as well as from E78 and E81 (forming the internal iron-binding motif) to the exposed K108 is shown.
(TIF)

Figure S4 Binding of haem by HbpS proteins. 20 µM of HbpS proteins (wild-type and the mutant E78A/E81A) were incubated with 10 µM haemin as described previously [35]. Haem binding was monitored spectrophotometrically. Spectra in the region between 375 to 450 nm are shown. The characteristic Soret peak at 411 nm is indicated by the arrow.
(TIF)

Table S1 List of plasmids and their relevant characteristics.
(DOC)

Table S2 Primers used to obtain the different HbpS mutants.
(DOC)
