# Supplementary figures and images for: Iron Binding at Specific Sites within the Octameric HbpS Protects Streptomycetes from Iron-Mediated Oxidative Stress
Source: PLoS One. 2013 Aug 27;8(8):e71579. doi: 10.1371/journal.pone.0071579 (PMC3754957; doi:10.1371/journal.pone.0071579)

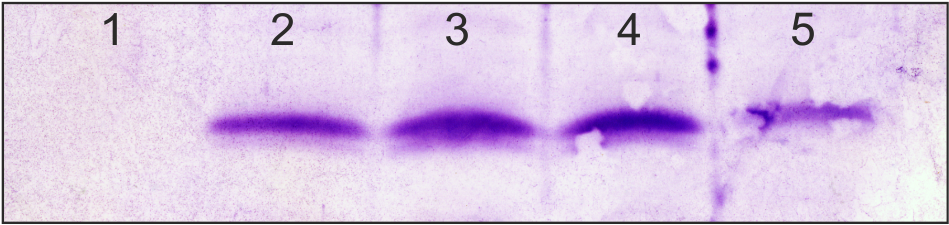

Supplement: Figure S1 — HbpS proteins are secreted in studied S. lividans tranformants. Culture filtrates of S. lividans transformants containing either pWHbpS-WT (lane 2) or pWHbpS-E78A (lane 3) or pWHbpS-E81A (lane 4) or pWHbpS-E78A/E81A (lane 5) or pWHM3 (lacking hbpS; lane 1) were subjected to Western blot analysis using anti-HbpS antibodies. (TIF) [file pone.0071579.s001.tif]

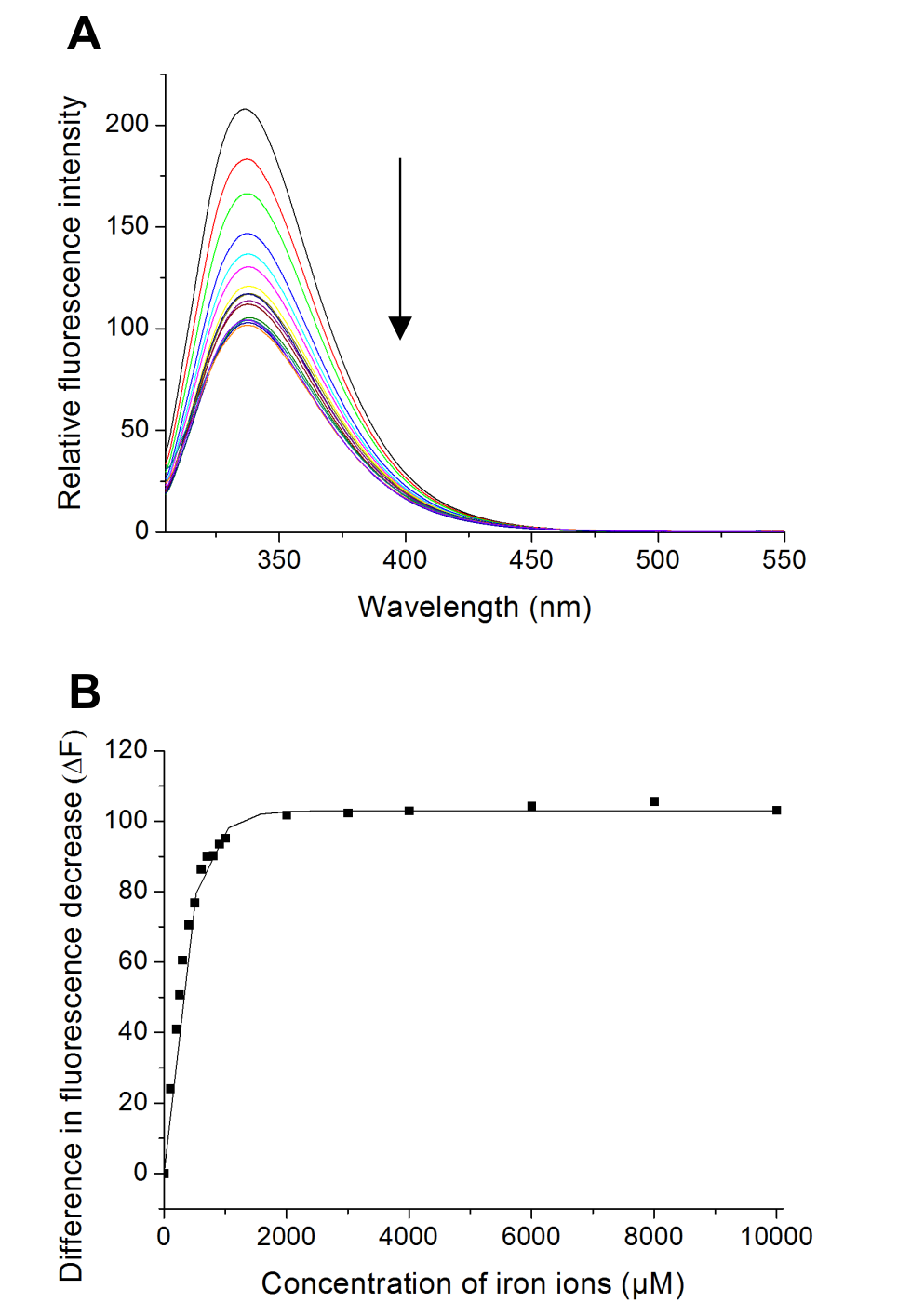

Supplement: Figure S2 — Iron-based Trp Fluorescence quenching of HbpS. (A) Fluorescence spectra of HbpS previously incubated with increasing concentrations (up to 10 mM; marked by the arrow) of ferrous iron ions. B) Differences in fluorescence (ΔF) were plotted against the concentration of titrated ferrous iron. (TIF) [file pone.0071579.s002.tif]

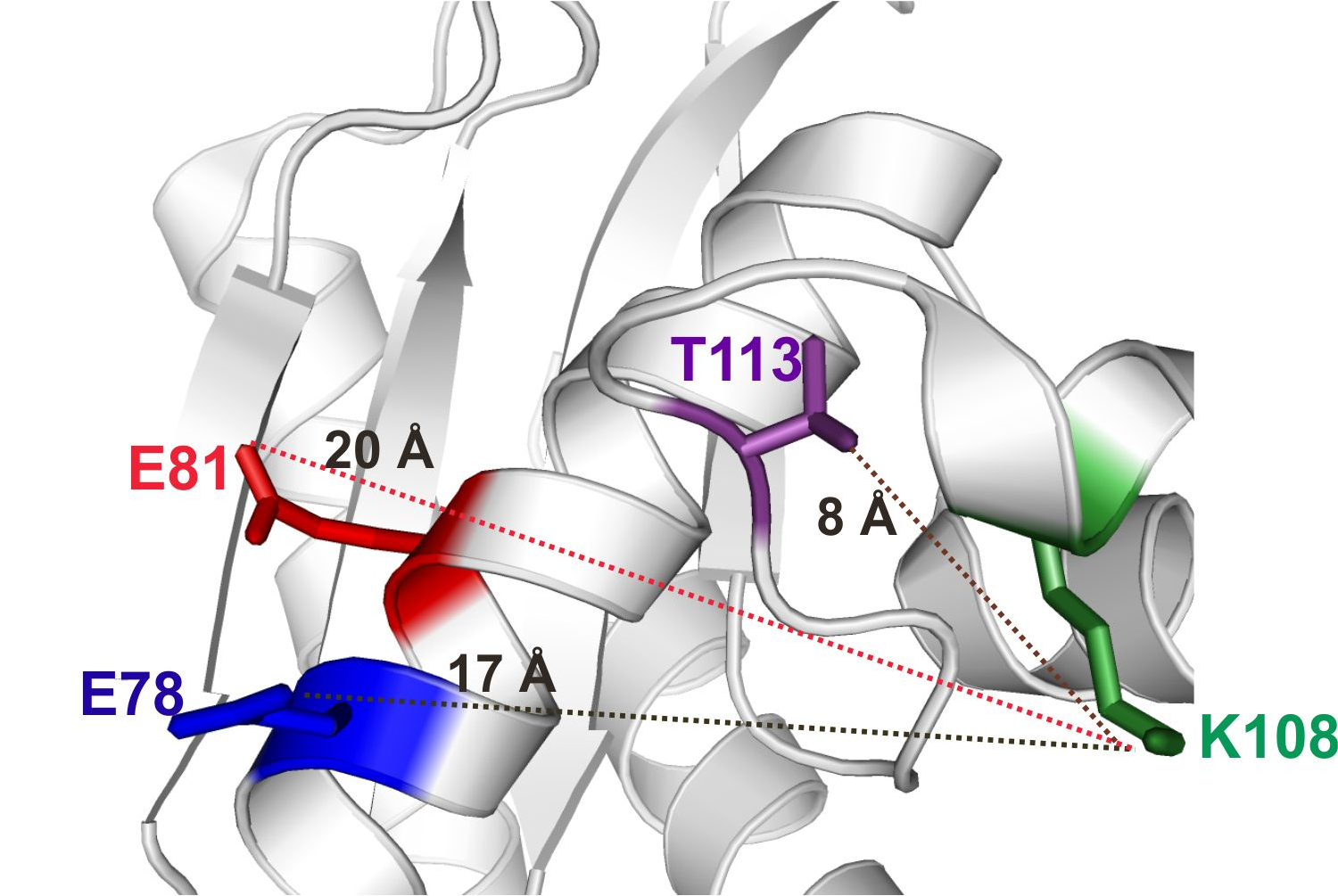

Supplement: Figure S3 — Arrangement of the haem-binding and some of the iron-binding sites within HbpS. The distance (in Å) from the haem-binding site at T113 as well as from E78 and E81 (forming the internal iron-binding motif) to the exposed K108 is shown. (TIF) [file pone.0071579.s003.tif]

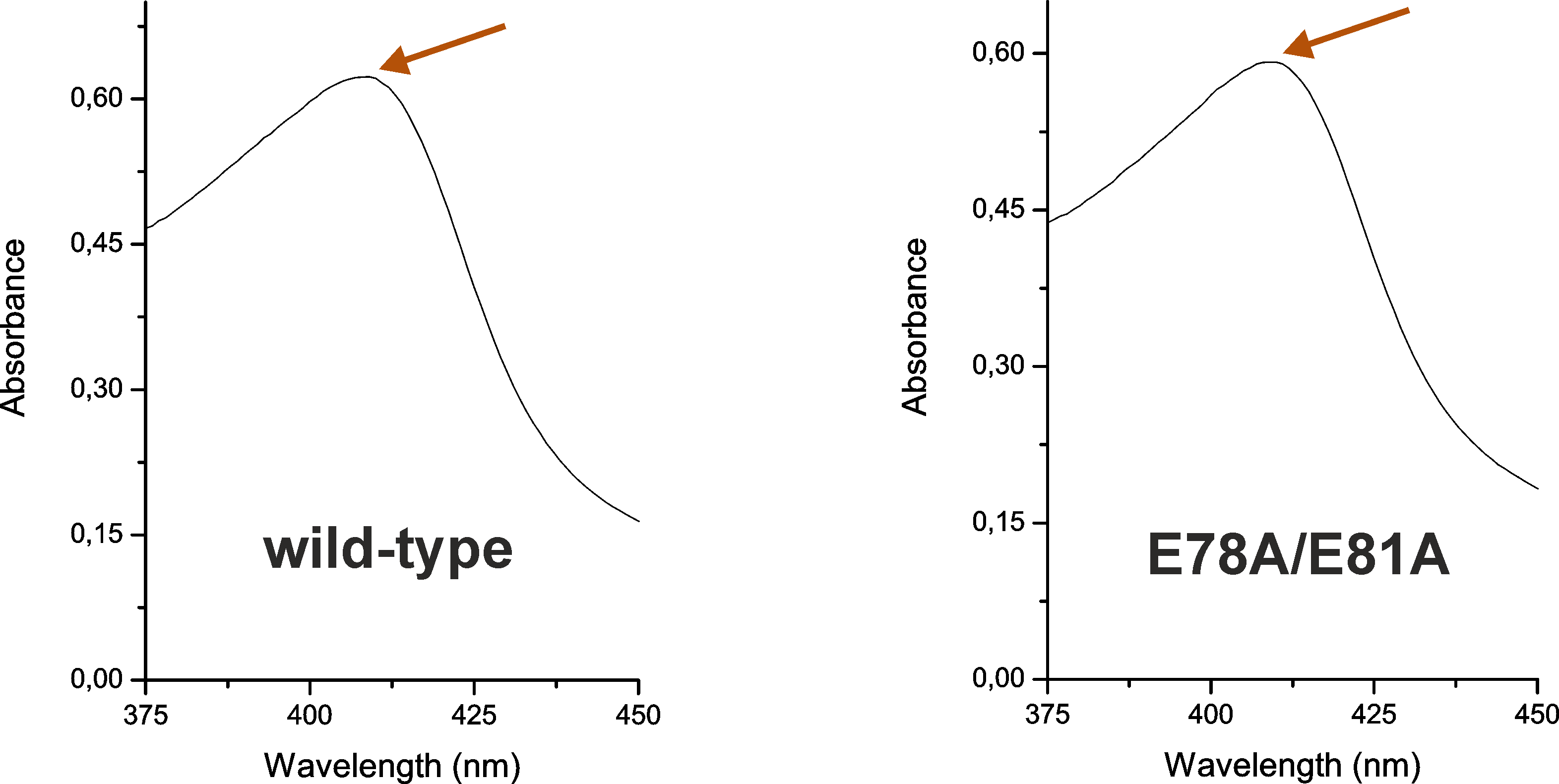

Supplement: Figure S4 — Binding of haem by HbpS proteins. 20 µM of HbpS proteins (wild-type and the mutant E78A/E81A) were incubated with 10 µM haemin as described previously [35]. Haem binding was monitored spectrophotometrically. Spectra in the region between 375 to 450 nm are shown. The characteristic Soret peak at 411 nm is indicated by the arrow. (TIF) [file pone.0071579.s004.tif]
