# Supplementary material for: Iron Binding at Specific Sites within the Octameric HbpS Protects Streptomycetes from Iron-Mediated Oxidative Stress
Source: PLoS One. 2013 Aug 27;8(8):e71579. doi: 10.1371/journal.pone.0071579 (PMC3754957; doi:10.1371/journal.pone.0071579)
Supplement: Table S1 — List of plasmids and their relevant characteristics. (DOC) [file pone.0071579.s005.doc]

| **Plasmid** | **Characteristics** | **Source** |
| --- | --- | --- |
| pETHbpS | containing *hbpS* without the sequence for the signal peptide with six | [43] |
|  | histidine codons at the 5' end and a TEV protease cleavage site |  |
| pETHbpS-E78A | pETHbpS with *hbpS* containing an ala codon instead of Glu | This work |
| pETHbpS-E81A | pETHbpS with *hbpS* containing an ala codon instead of glu | This work |
| pETHbpS- E78A/E81A | pETHbpS with *hbpS* containing two ala codons instead of two glu | This work |
| pETHbpS-E43A | pETHbpS with *hbpS* containing an ala codon instead of glu | This work |
| pETHbpS-E46A | pETHbpS with *hbpS* containing an ala codon instead of glu | This work |
| pETHbpS- E43A/E46A | pETHbpS with *hbpS* containing two ala codons instead of two glu | This work |
| pETHbpS-K108A | pETHbpS with *hbpS* containing an ala codon instead of lys | [36] |
| pETHbpS-K83A | pETHbpS with *hbpS* containing an ala codon instead of lys | This work |
| pETHbpS-K83R | pETHbpS with *hbpS* containing an arg codon instead of lys | This work |
| pETHbpS-R82A | pETHbpS with *hbpS* containing an ala codon instead of arg | This work |
| pETHbpS-H28A | pETHbpS with *hbpS* containing an ala codon instead of his | [36] |
| pETHbpS-E78D | pETHbpS with *hbpS* containing an asp codon instead of glu | This work |
| pETHbpS-E81D | pETHbpS with *hbpS* containing an asp codon instead of glu | This work |
| pETHbpS- E78D/E81D | pETHbpS with *hbpS* containing two asp codons instead of two glu | This work |
| pETHbpS-D141A | pETHbpS with *hbpS* containing an ala codon instead of asp | This work |
| pETHbpS-D143A | pETHbpS with *hbpS* containing an ala codon instead of asp | This work |
| pETHbpS-E144A | pETHbpS with *hbpS* containing an ala codon instead of glu | This work |
| pETHbpS-Y77F | pETHbpS with *hbpS* containing an phe codon instead of tyr | [38] |
| pETHbpS-Y77A | pETHbpS with *hbpS* containing an ala codon instead of tyr | This work |
| pUKS10 | pUBB1 derivative containing *furS, cpeB, hbpS* and *bla* | [46] |
| pUKS13 | pUKS10 derivative containing the *furS-cpeB* operon with its promoter and | [45] |
|  | mutated *furS*; mutation in *furS* leads to an inactive repressor |  |
| pUKS20 | *Eco*RI-*Stu*I fragment of pUKS13 and *Eco*RI-*Sma*I fragment of pUC18 | This work |
| pUKS21 | pUKS20 with introduced *Nco*I restriction site between *furS* and *cpeB* | This work |
| pUKS22 | pUKS21 with *hbpS* from pUKS10 resulting in a *furS-hbpS* operon | This work |
| pUKS23 | pUKS22 derivative with *hbpS* containing an ala codon instead of a glu | This work |
|  | codon (E78A) |  |
| pUKS24 | pUKS22 derivative with *hbpS* containing an ala codon instead of a glu | This work |
|  | codon (E81A) |  |
| pUKS25 | pUKS22 derivative with *hbpS* containing two ala codons instead of two | This work |
|  | glu codons (E78A/E81A) |  |
| pWHM3 | multiple cloning site, *bla* and *tsr*^r^ | [66] |
| pWHbpS | pWHM3 derivative containing the *Eco*RI-*Hin*dIII fragment from pUFHbpS | This work |
|  | coding for *furS* and *hbpS* |  |
| pWHbpS-E78A | pWHbpS with *hbpS* containing an ala codon instead of glu | This work |
| pWHbpS-E81A | pWHbpS with *hbpS* containing an ala codon instead of glu | This work |
| pWHbpS- E78A/E81A | pWHbpS with *hbpS* containing two ala codons instead of two glu | This work |
