# Supplementary material for: Iron Binding at Specific Sites within the Octameric HbpS Protects Streptomycetes from Iron-Mediated Oxidative Stress
Source: PLoS One. 2013 Aug 27;8(8):e71579. doi: 10.1371/journal.pone.0071579 (PMC3754957; doi:10.1371/journal.pone.0071579)
Supplement: Table S2 — Primers used to obtain the different HbpS mutants. (DOC) [file pone.0071579.s006.doc]

| **Primer** | **Sequence (5' → 3')** |
| --- | --- |
| PET11Rev | GGGCTTTGTTAGCAGCCGGATCTCAG |
| PET11FOR | GTGAGCGGATAACAATTCCCCTCTAG |
| PEAFor | CAGTCGTACGCCTCCGCGGCGCGCAAGGCGTTCAC |
| PE78A | CAGTCGTACGCCTCCGCGGAGCGCAAGGC |
| PE81A | CAGTCGTACGAGTCCGCGGCCCGCAAGGCGTTCACCGC |
| PR82A | CAGTCGTACGAGTCCGCGGAGGCCGCGGCGTTCACCGCG |
| PRevE43A | CCGACACGTGCCGGCCGTCCTTCTCGGCCCCCGCCACCGCGGGCGC |
| PFor46A | GCGGCCGCGAAGGACGGCCGGCACG |
| PRev43-46 | CTTCGCGGCCGCCGCCACCGCGGCGCGCGCC |
| PK83A | CAGTCGTACGAGTCCGCGGAGCGCGCGGCGTTCACCGCGGTGTC |
| PK83R | CAGTCGTACGAGTCCGCGGAGCGCAGGGCGTTCACCGCGGTGTC |
| PE78D | CAGTCGTACGACTCCGCGGAGCGCAAGGC |
| PE81D | CAGTCGTACGAGTCCGCGGACCGCAAGGCGTTCACCGC |
| PEEDD | CAGTCGTACGACTCCGCGGACCGCAAGGCGTTCACCCGC |
| PRev46A | GCCGTCCTTCGCGGCCGCCTCCACCG |
| PFor43-46 | GGTGGCGGCGGCCGCGAAGGACGGCCGGCACG |
| PD141ARev | CCAAGCTTTCAGTGGCCGAGCACGGCCGCGCCCGCCCGTGCGTAC |
|  | TGCTCGTCCAGGGCGCCCGACGGAGCAC |
| PE144ARev | GCAAGCTTTCAGTGGCCGAGCACGGCCGCGCCCGCCCGTGCGTAC |
|  | TGCGCGTCCAGGTCGCCCG |
| PANotFor | CGCGCGGCCGCGGCCGGTCCGGGGCC |
| PANcoRev | CAGCCATGGCAATCCTTCCGAACTGTGC |
| PBNcoFor | GATTGCCATGGCTGAGAACCACGACGCG |
| PBHinRev | GTGCCAAGCTTGACTGCCTGCAGGTCG |
| PHbpFor | GTCAGCATGGCCTCCAGCCGCAAGAAG |
| PHbpRev | CAGAAGCTTTCAGTGGCCGAGGAC |
| PYAFor2 | GTCGGCGGAGTCCGCGGAGCGCAAG |
| PYARev1 | GACTCCGCCGACTGCGGGCCCGCCCCG |
